# The Structure of Borders in a Small World

**DOI:** 10.1371/journal.pone.0015422

**Published:** 2010-11-18

**Authors:** Christian Thiemann, Fabian Theis, Daniel Grady, Rafael Brune, Dirk Brockmann

**Affiliations:** 1 Department of Engineering Sciences and Applied Mathematics, Northwestern University, Evanston, Illinois, United States of America; 2 Max Planck Institute for Dynamics and Self-Organization, Göttingen, Germany; 3 Institute for Bioinformatics and Systems Biology, Helmholtz Center Munich, Neuherberg, Germany; 4 Department of Mathematics, University of Technology Munich, Garching, Germany; 5 Northwestern Institute on Complex Systems, Evanston, Illinois, United States of America; Cajal Institute, Consejo Superior de Investigaciones Científicas, Spain

## Abstract

Territorial subdivisions and geographic borders are essential for understanding phenomena in sociology, political science, history, and economics. They influence the interregional flow of information and cross-border trade and affect the diffusion of innovation and technology. However, it is unclear if existing administrative subdivisions that typically evolved decades ago still reflect the most plausible organizational structure of today. The complexity of modern human communication, the ease of long-distance movement, and increased interaction across political borders complicate the operational definition and assessment of geographic borders that optimally reflect the multi-scale nature of today's human connectivity patterns. What border structures emerge directly from the interplay of scales in human interactions is an open question. Based on a massive proxy dataset, we analyze a multi-scale human mobility network and compute effective geographic borders inherent to human mobility patterns in the United States. We propose two computational techniques for extracting these borders and for quantifying their strength. We find that effective borders only partially overlap with existing administrative borders, and show that some of the strongest mobility borders exist in unexpected regions. We show that the observed structures cannot be generated by gravity models for human traffic. Finally, we introduce the concept of link significance that clarifies the observed structure of effective borders. Our approach represents a novel type of quantitative, comparative analysis framework for spatially embedded multi-scale interaction networks in general and may yield important insight into a multitude of spatiotemporal phenomena generated by human activity.

## Introduction

The geographic compartmentalization of maps into coherent territorial units is not only essential for the management and distribution of administrative responsibilities and the allocation of public resources. Territorial subdivisions also serve as an important frame of reference for understanding a variety of phenomena related to human activity. Existing borders frequently correlate with cultural and linguistic boundaries or topographical features [1], [2], they represent essential factors in trade and technology transfer [3], [4], and they indirectly shape the evolution of human-mediated dynamic processes such as the spread of emergent infectious diseases [5]–[8].

The majority of existing administrative and political borders, for example in the United States and Europe, evolved over centuries and typically stabilized many decades ago, during a time when human interactions and mobility were predominantly local and the conceptual separation of spatially extended human populations into a hierarchy of geographically coherent subdivision was meaningful and plausible.

However, modern human communication and mobility has undergone massive structural changes in the past few decades [1], [9]. Efficient communication networks, large-scale and widespread social networks, and more affordable long-distance travel generated highly complex connectivity patterns among individuals in large-scale human populations [10], [11]. Although geographic proximity still dominates human activities, increasing interactions over long distances [12]–[14] and across cultural and political borders amplify the small-world effect [15], [16] and decrease the relative importance of local interactions.

### Multi-Scale Human Mobility

**Figure 1:**
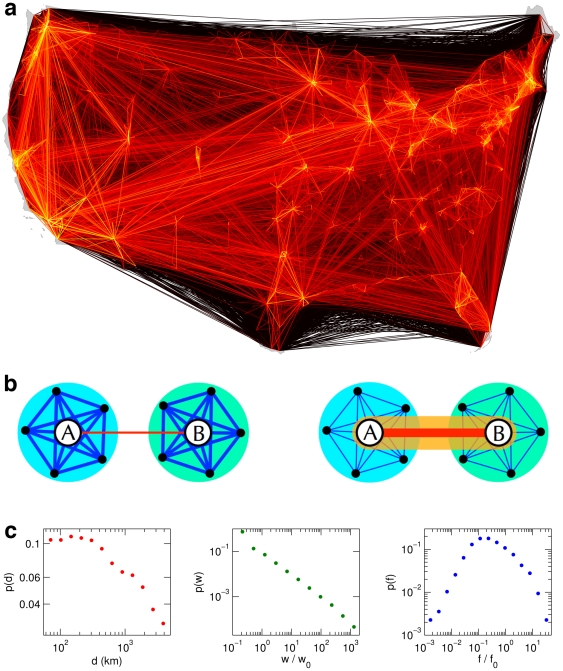
Human mobility network derived from bank note fluxes. (*a*) Multi-scale human mobility is characterized by dominant short range and significant long-range connectivity patterns. The illustrated network represents a proxy for human mobility, the flux of bank notes between 3,109 counties in the lower 48 United States. Each link 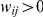 is represented by a line, the color scale encodes the strength of a connection from small (dark red) to large (bright yellow) values of 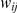 spanning four orders of magnitude. (*b*) A simplified illustration of generic traffic patterns between and within metropolitan mobility hubs (A and B), with two types of connections 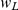 and 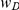, local traffic connecting individual hubs to smaller nodes in their local environment (blue) and long distance links connecting the hubs (red). Depending on the ratio of local and long range flux magnitude, two qualitatively different modularizations are plausible. If 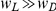, two spatially compact communities are meaningful (left), whereas if 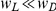, the metropolitan centers belong to one yet geographically delocalized module (orange), effectively detached from their local environment, yielding three communities altogether (right). (*c*) Multi-scale mobility networks are strongly heterogeneous as reflected by the functions 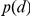, 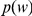, and 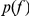, the relative frequencies of distances 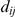, link weights 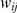 and vertex flux 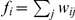 that all are distributed over several orders of magnitude.

Human mobility networks epitomize the complexity of multi-scale connectivity in human populations (see Fig. 1a). More than 17 million passengers travel each week across long distances on the United States air transportation network alone. However, including all means of transportation, 80% of all traffic occurs across distances less than 50 km [12], [17]. The coexistence of dominant short-range and significant long-range interactions handicaps efforts to define and assess the location and structure of effective borders that are implicitly encoded in human activities across distance. The paradigm of spatially coherent communities may no longer be plausible, and it is unclear what structures emerge from the interplay of interactions and activities across spatial scales [12], [13], [17], [18]. This difficulty is schematically illustrated in Fig. 1b. Depending on the ratio of local versus long-range traffic, one of two structurally different divisions of subpopulations is plausible. If short-range traffic outweighs long-range traffic, local, spatially coherent subdivisions are meaningful. Conversely, if long-range traffic dominates, subdividing into a single, spatially de-coherent urban community and disconnected suburban modules is appropriate and effective geographic borders are difficult to define in this case.

Although previous studies identified community structures in long-range mobility networks based on topological connectivity [19], [20], this example illustrates that the traffic intensity resulting from the interplay of mobility on all spatial scales must be taken into account. Obtaining comprehensive, complete, and precise datasets on human mobility covering many spatial scales is a difficult task, and recent studies have followed a promising alternative strategy based on the analysis of proxies that permit the indirect measurement of human mobility patterns [9], [12]–[14], [21], [22].

## Results and Discussion

### A Proxy for Human Mobility

Here we construct a proxy network for human mobility from the geographic circulation of banknotes in the United States. Movement data was collected using the online bill-tracking game wheresgeorge.com. Individuals participating in this game can mark individual bills and return them to circulation; other individuals who randomly receive bills can report this find online along with their current location (zip code). A comprehensive account of the circulation of currency, the wheresgeorge.com dataset, and an analysis of the statistics of travel distances and inter-report times for various denominations are provided in Text S1. Our analysis is based on the intuitive notion that the coupling strength between two locations 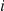 and 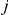 increases with 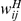, the number of individuals that travel between a pair of locations per unit time, and furthermore that the flux of individuals in turn is proportional to the flux of bank notes, denoted by 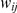. Evidence for the validity of this assumption has been obtained previously [12], [13], [17] and we provide further evidence in Text S1. Based on a set of trajectories of 11,759,420 bills we thus compute 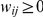 between the 3,109 counties in the lower 48 United States (excluding Alaska and Hawaii). The resulting proxy network for human mobility is thus encoded by the matrix 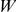 whose elements are 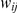. 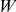 represents a symmetric, weighted, spatially-embedded network. The network is multi-scale, ranging from the typical linear extent of a county (approximately 50 km) to the linear size of the US (approximately 4,000 km), and strongly heterogeneous, reflected in the broad distribution of the weights, degrees, and the fluxes per node (cf. Fig. 1c, video clip at http://rocs.northwestern.edu/clips/?assets/Follow_the_Money_SD.mp4).

### Effective Geographic Subdivisions and Borders

Based on the idea that two counties 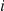 and 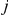 are effectively proximal if 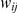 is large, we use network-theoretic techniques [23] to identify a partition 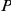 of the nodes into 
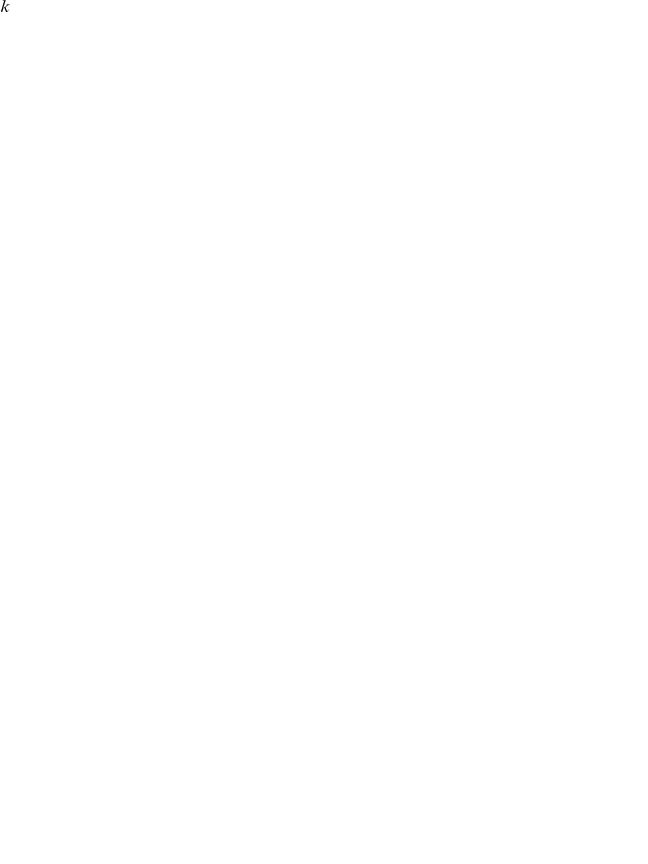
 modules 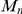 such that the intra-connectivity of the modules in the partition is high and inter-connectivity between them is low as compared to a random null model. A standard measure of the amount of community structure captured by a given partition 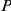 is the modularity 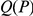 defined as [24], [25]

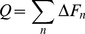
(1)in which 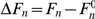 is the difference between 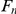, the fraction of total mobility within the module 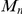, and the expected fraction 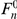 of a random network with an identical weight distribution 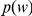. 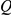 cannot exceed unity; high values indicate that a partition successfully groups nodes into modules, whereas random partitions yield 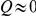. Maximizing 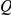 in large networks is an NP-hard problem [26], but a variety of algorithms have been developed to systematically explore and sample the space of possible divisions in order to identify high-modularity partitions [23], [27].

#### Modularity Maximization

**Figure 2:**
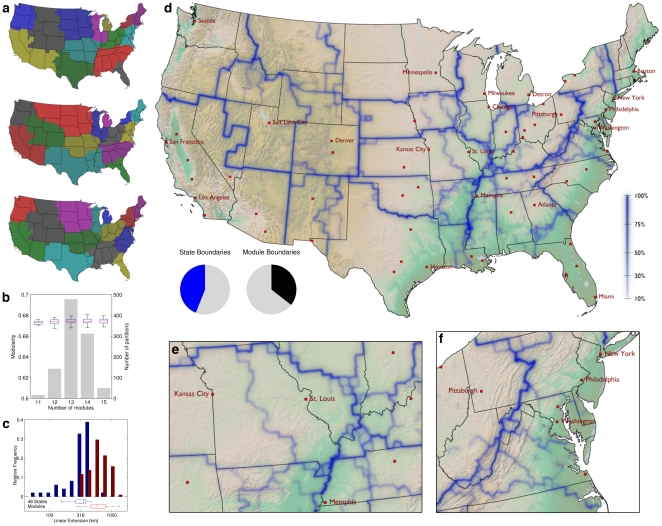
Effective subdivisions and borders in the United States. (*a*) Subdivisions determined by maximizing modularity 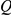 share similar values of 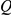 (top to bottom: 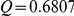, 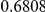, and 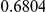, all in 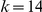 modules). In all maps the modules are spatially compact. Although these solutions share features, they exhibit significant differences in the module structure. (*b*) Ensemble statistics of geographic subdivisions for a set of 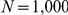 partitions. The number of modules 
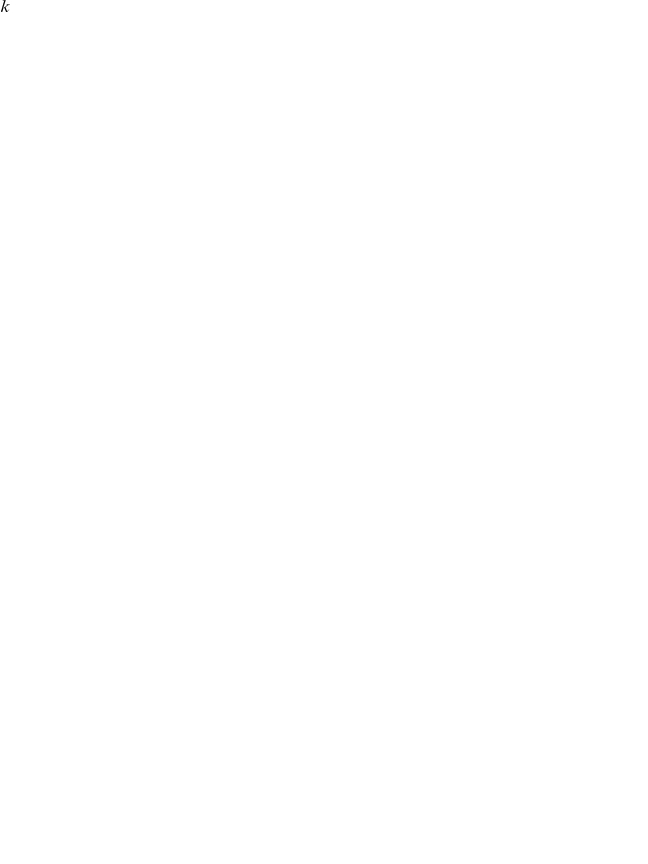
 in each subdivision is narrowly distributed around 13 (grey bars), and so are the conditional distributions of modularity (superimposed whisker plots). The ensemble mean is 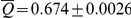. (*c*) Distribution of the linear extensions of the 48 states (mean 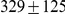 km) and the geographic modules in the effective subdivision (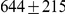 km). (*d*) Effective borders emerge from linear superposition of all maps in the ensemble (blue lines). Intensity encodes border significance (i.e. the fraction of maps that exhibit the border). Black lines indicate state borders. Although 44% of state borders coincide with effective borders (left pie chart), approximately 64% of effective borders do not coincide with state borders. These borders are statistically significant features of the ensemble of high modularity maps, they partially correlate with administrative borders, topographical features, and frequently split states. (*e*) Close-up on the Missouri region, showing the effective border between Kansas City and St. Louis that divides the state. (*f*) Close-up on the Appalachian Mountains with corresponding border, which extends north to split Pennsylvania. This border is the strongest in the map.

We employ a stochastic Monte-Carlo method (see Text S1, video clip at http://rocs.northwestern.edu/clips/?assets/Follow_the_Money_SD.mp4) to approximate a maximal-modularity subdivision of the multi-scale mobility network depicted in Fig. 1a. Since the optimization process is stochastic, the resulting partition varies between realizations of the process. Three representative examples of high-modularity partitions are displayed in Fig. 2a. Note that, although modularity only takes into account the structure of the weight matrix 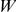 and is explicitly blind to the geographic locations of nodes, the effective large-scale modules are spatially compact in every map. Consequently, although long-distance mobility plays an important role, the massive traffic along short distances generates spatial coherence of community patches of mean linear extension 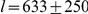 km. Note however that although each maps exhibits qualitative similarities between detected large scale subdivisions and although each of the maps possess a high modularity score, obvious structural differences exist. It is thus questionable whether one individual effective maps can be considered the single most plausible partition. Recent work has moreover identified a resolution limit intrinsic to the modularity measure that renders modularity-maximization algorithms incapable of detecting modules below a critical size [28].

#### Assessment of Border Structures

Therefore, instead of focusing on a single high-modularity map, we consider an entire ensemble of partitions that can be exploited to recover the underlying community structure and alleviate the aforementioned resolution limit (see Text S1). We compute an ensemble of 1,000 partitions, all exhibiting a high modularity (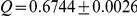) and spatially compact modules, and perform a linear superposition of the set of maps. This method extracts features that are structural properties of the entire ensemble. The most prominent emergent feature is a complex network of spatially continuous geographic borders (Fig. 2d). These borders are statistically significant topological features of the underlying multi-scale mobility network. An important aspect of this method is the ability to not only identify the location of these borders but also to quantify the frequency with which individual borders appear in the set of partitions, a measure for the strength of a border (see video clip at http://rocs.northwestern.edu/clips/?assets/Follow_the_Money_SD.mp4).

Investigating this system of effective mobility borders more closely, we see that although they correlate significantly with territorial state borders (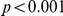, see Text S1) they frequently occur in unexpected locations. For example, they effectively split some states into independent patches, as with Pennsylvania, where the strongest border of the map separates the state into regions centered around Pittsburgh and Philadelphia. Other examples are Missouri, which is split into two halves, the eastern part dominated by St. Louis (also taking a piece of Illinois) and the western by Kansas City, and the southern part of Georgia, which is effectively allocated to Florida. Also of note are the Appalachian mountains. Representing a real topographical barrier to most means of transportation, this mountain range only partially coincides with state borders, but the effective mobility border is clearly correlated with it. Finally, note that effective patches are often centered around large metropolitan areas that represent hubs in the transportation network, for instance Atlanta, Minneapolis and Salt Lake City. We find that 44% of the administrative state borders are also effective boundaries, while 64% of all effective boundaries do not coincide with state borders (cf. pie charts in Fig. 2d).

### Understanding Effective Borders

**Figure 3:**
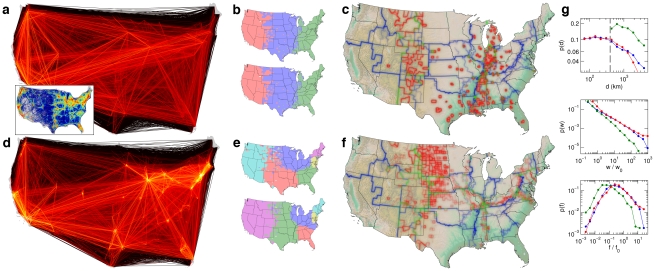
Comparative analysis of effective borders in two artificial systems : a modified mobility network deprived of short distance traffic (a–c), and a gravity model for human mobility (d–f). (*a*) A subnetwork of the original system (Fig. 1a) in which all links with geographic length 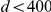 km are removed (the inset depicts the complementary, removed subnetwork). (*b*) Two generic partitions of this long-range network, consisting of only three modules that do not exhibit sharply defined geographical borders. (*c*) The resulting border structure (red lines) exhibits no significant overlap with the borders obtained from the original multi-scale system. Borders of the original system and overlap are depicted in blue and green, respectively. (*d*) A gravity model network as defined by Eq. (2). Parameters 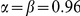 and 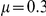 have been chosen to maximize first-order statistical similarity to the original data. (*e*) Although qualitatively the network in (d) shares features with the original network (Fig. 1a), generic partitions of the gravity model network are structurally different, typically exhibiting fewer modules per partition, in different locations and with less spatial compactness. (*f*) The border structure of the gravity network (red) partially coincides with the borders in the original data (blue), but not significantly. The overlap is shown in green, for significance tests see Text S1. (*g*) First order statistics of the two artificial networks in comparison to the original network. The functions 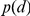, 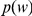, and 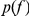 for the long-range network in (a) (green), the gravity model network in (d) (red), and the original mobility network (Fig. 1a, blue). The dotted line indicates 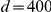 km.

A key question is what components of the network are responsible for the features observed in the system of effective borders. In order to test the degree to which short-range connections dominate the structure of effective borders we generate an artificial network that lacks short-range connections (Fig. 3a). Applying the same computational technique to locate and quantify effective spatial subdivisions, we find that removing short-distance traffic has profound consequences for the spatial structure and coherence of divisions. We consistently find three independent modules that latitudinally split the US. As these three modules remain largely spatially coherent, we conclude that intermediate traffic inherits the role of short range mobility in generating spatial coherence. Although the removal of short links represents a substantial modification of the network, bootstrapping the original network randomly by the same amount (see Text S1) has little impact on the border structure depicted in Fig. 2d. We conclude that short- to intermediate-distance mobility is a key factor in shaping effective borders.

#### Comparison to Gravity Models

We also investigate whether the observed pattern of borders can be accounted for by the prominent class of gravity models [29]–[31], frequently encountered in modeling spatial disease dynamics [31]. In these phenomenological models it is assumed that the interaction strength 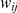 between a collection of sub-populations with geographic positions 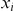, sizes 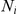, and distances 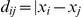 is given by
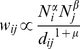
(2)in which the exponents 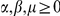 are parameters. Although their validity is still a matter of debate, gravity models are commonly used if no direct data on mobility is available. The key feature of a gravity model is that 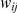 is entirely determined by the spatial distribution of sub-populations. We therefore test whether the observed patterns of borders (Fig. 2d) are indeed determined by the existing multi-scale mobility network or rather indirectly by the underlying spatial distribution of the population in combination with gravity law coupling. Figure 3f illustrates the borders we find in a network that obeys equation (2). We generate this network such that the first order statistical similarity to the original networks is maximized, which sets the parameters 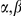 and 
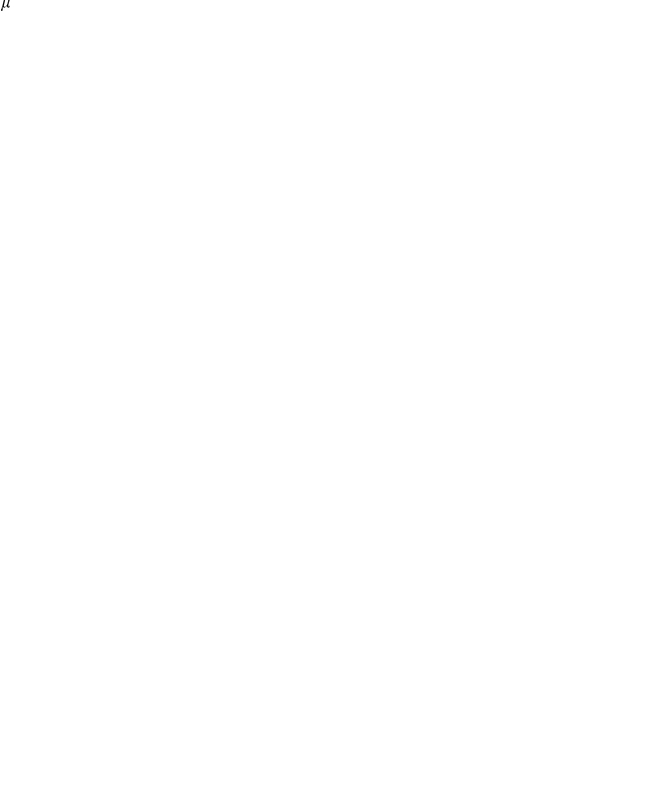
 (see Text S1). Comparing this model network to the original multi-scale network we see that their qualitative properties are similar, with strong short range connections as well as prominent long range links. However, maximal modularity maps typically contain only five subdivisions with a mean modularity of only 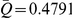. Because borders determined for the model system are strongly fluctuating (maps in Fig. 3e), they yield much less coherent large scale patches. However, some specific borders, e.g. the Appalachian rim, are correctly reproduced in the model. Because the model system produces significantly different patterns (see Text S1 for statistics), we conclude that the sharp definition of borders in the original multi-scale mobility network and the pronounced spatial coherence of the building blocks are an intrinsic feature of the real multi-scale mobility network and can not be generated by a gravity model that has a maximum first order statistical overlap with the original mobility network.

#### Effective Borders and Shortest Path Trees

**Figure 4:**
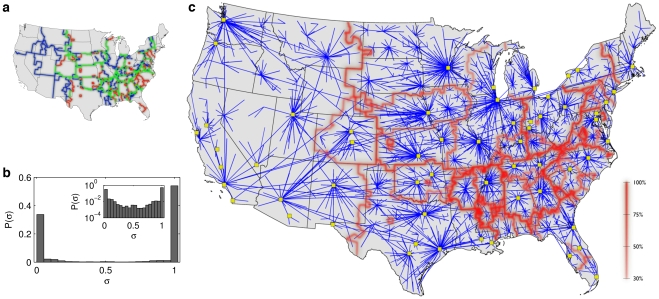
The relation of effective borders and the significant links of the multi-scale mobility network. (*a*) Comparing borders from modularity maximization (blue) with SPT clustering (red) reveals a significant overlap (green). The cumulative topological overlap (see Text S1) is 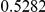 indicating that the SPTD method represents an alternative computational approach to border extraction. (*b*) The distribution of link significance 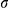, defined for each link as the number of shortest-path trees the link appears in, exhibits a strong bimodal distribution. This implies that SPTD can sort links into important or not, and that 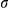 is approximately a binary variable. (*c*) By comparing the border structure from SPT clustering with the ensemble of significant links (those that appear in at least half of the shortest-path trees, i.e. in the right half of the histogram in *b*) we identify topological structures which reveal the core of the network that explains the majority of border locations. This core is represented by the network in blue consisting of star-shaped modules centered around large cities (yellow squares).

The proposed method successfully extracts the structure of geographic borders inherent in multi-scale mobility networks. Bootstrapping the network indicates that these structures are surprisingly stable in response to perturbations of the network, but neither the modularity measure nor the stochastic algorithm we use to discover partitions provide specific information about the substructures in the network that make these borders so robust. What feature of the network, more specifically which subset of links if any, generates the observed borders? In order to address this question and further investigate the structural stability of the observed patterns, we developed a new and efficient computational technique based on the concept of shortest-path trees (SPT) [32]. Like stochastic modularity maximization, this technique identifies a structure of borders that encompass spatially coherent regions (Fig. 4c), but unlike modularity this structure is unique. More importantly, it identifies a unique set of connections in the network, a network backbone, that correlates strongly with the observed borders. The shortest-path tree 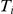 rooted at node 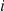 is the union of all shortest paths originating at 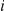 and ending at other nodes. The shortest path between two nodes is the path that minimizes the effective distance 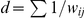 along the legs of the path. Based on the set of SPTs 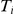 we compute an effective distance between nodes 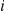 and 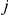 by computing the shortest path tree dissimilarity (SPTD), i.e.
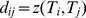
(3)Details of the function 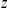 that quantifies differences of trees are provided in Text S1. If 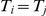 we have 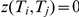, whereas 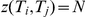 for completely different trees. In our data the 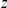 values range from 2 to 240. We compute a series of borders induced by tree dissimilarity by applying a standard hierarchical clustering algorithm [33] to the complete dissimilarity matrix 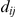. We consider a border more important if it appears earlier in the hierarchy. Unlike conventional clustering of the inverse weight matrix which requires adding some noise and produces a hierarchical structure that does not strongly correlate with the input, the set of borders computed by our method is an accurate representation of the underlying data (see Text S1). In fact, although the method yields a unique sequence of topological segmentations, the observed geographic borders exhibit a strong correlation with those determined by modularity maximization (Fig. 4a).

The key advantage of this method is that it can systematically extract properties of the network that match the observed borders. A way to demonstrate this is to measure the frequency 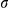 at which individual links appear in the ensemble of all SPTs, which is conceptually related to their link betweenness [25]. Computing this *link significance* 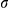 for each connection, we find that the distribution 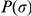 of the network is bimodally peaked (Fig. 4b). This is a promising feature of 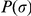 as it allows labeling links as either significant or redundant without introducing an arbitrary threshold which is necessary for more continuously distributed link centrality measures. Extracting the group of significant links and constructing a subnetwork from these links only we observe that this subnetwork matches the computed border structure (Fig. 4c). By virtue of the fact that the most frequently shared links between SPTs are local, short-range connections we see that the SPT boundaries enclose local neighborhoods and that the boundaries fall along lines where SPTs do not share common features. Note that effective metropolitan areas around cities can be detected with greater precision than modularity, although the western US is detected as effectively a single community.

Finally, we performed statistical analyses that quantify the overlap of the effective, mobility-induced borders with those provided by census-related systems. We choose the set of borders separating the states, the borders defined by the districts of the 12 Federal Reserve Banks, and the borders of Economic Areas [34]. Details of this correlation analysis are provided in Text S1. We find a significant correlation with economic boundaries (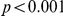, 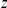-score 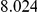 for the modularity borders and 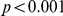, 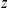-score 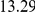 for the SPT borders).

We conclude that considerable geographic information is not only effectively encoded in human mobility networks, it can also be identified systematically using the techniques presented here. The identification and quantification of geographic borders and a comprehensive assessment of their significance will be very important for understanding dynamic processes driven by human mobility. An important area in which the observed borders potentially facilitate our understanding is the geographic patterns of isoglosses in North American English dialects [35]. Partial evidence exists that isoglosses correlate with mobility patterns. The results presented here may serve as a starting point for a better understanding of language borders not only in North American English but generally in the context of spatial linguistics.

Although applied here to a network that spans a continent, the proposed concepts are quite general and can be applied on a finer geographical scale. This could lead to new methods for urban design and for planning public transportation systems. The ideas need not be limited to human behavior: tracking of various animals is now widespread, and a dataset of sufficient size, in combination with these techniques, could help understand animal foraging and habitat segmentation. The framework presented here is suitable for a wide range of multi-scale interaction networks for which the underlying effective borders are presently unknown. We believe that our discovery of effective mobility borders in the US is a first step and that these techniques will open the door to promising, quantitative, comparative investigations of many spatially distributed behavioral patterns.

## Supporting Information

Text S1 (PDF)
