# Supplementary material for: The Structure of Borders in a Small World
Source: PLoS One. 2010 Nov 18;5(11):e15422. doi: 10.1371/journal.pone.0015422 (PMC2987795; doi:10.1371/journal.pone.0015422)
Supplement: Text S1 — (PDF) [file pone.0015422.s001.pdf]

The structure of borders in a small world: Text S1

October 19, 2010

Contents

1. The Where's George dataset **2**
1.1. Geographical distributions . . . 3
1.2. Distance and time: spatially averaged quantities . . . 5
1.3. Definition of the mobility network . . . 6

2. Validity of currency flux as a proxy for human mobility **8**
2.1. Time scale analysis . . . 8
2.2. Denomination analysis . . . 12

3. Ensembles of high-modularity partitions **14**
3.1. Finding optimal partitions . . . 14
3.2. Superposition of partitions and limitations of modularity . . . 14
3.3. Bootstrapping the Where's George data . . . 17

4. Gravity as a null model **19**

5. Shortest-path tree clustering **21**
5.1. Computing shortest-path trees . . . 21
5.2. Measuring tree distance . . . 21
5.3. Hierarchical clustering . . . 21

6. Significance and comparison of border structures **25**
6.1. Measuring overlap of two boundary networks . . . 25
6.2. Randomization of the mean partition boundary of the Where's George network . . . 28
6.3. Significances when comparing boundary networks with the null model . . . 28
6.4. Discussion . . . 29

A. Global network properties **31**

1. The Where's George dataset

As of January 15th, 2010 a total of $187,925,059$ individual bills are being tracked at the website `www.wheresgeorge.com`. Approximately 11.24% of those have had "hits", i.e. they were reported a second time at the site after initial entry. The current analysis is based on a set of $N_0 = 11,950,239$ bills that were reported at least a second time. For each bill n we have a sequence of pairs of data

$$B_n = \{Z_{n,i}, T_{n,i}\} \quad i = 0, \ldots, L_n \quad n = 1, \ldots, N_0$$

of zip codes $Z_{n,i}$ and times $T_{n,i}$ at which the bill was reported. Each B_n reflects a geographic trajectory of a bill with L_n individual legs. In total, we have $14,612,391$ single legs in our database. Note that the majority (81.78%) of trajectories are single-legged reflecting a reporting probability of $\approx 20\%$ during the lifetime of a bill.

The set of B_n represents the core dataset of our analysis. For each bill we have additional information:

1. Denomination: \$1, \$2, \$5, \$10, \$20, \$50, or \$100. The fraction of each denomination is depicted in Table S1.

2. The Federal Reserve Bank code, A through L, corresponding to one of 12 of the United States Federal Reserve Banks that issued the bill. The fraction of bills as a function of FRB origin is provided in Table S2.

Table S1: Denominations in the WG dataset

Denomination	\$1	\$2	\$5	\$10	\$20	\$50	\$100
Number of bills	9,931,261	36,639	1,069,427	401,101	461,076	24,209	26,526
Fraction [%]	83.11	0.31	8.95	3.36	3.86	0.20	0.22

Table S2: Absolute number and relative fraction of bills based on Federal Reserve Bank

FRB Code	Location	Count	Fraction [%]
A	Boston	799,537	6.69
B	New York City	1,325,942	11.10
C	Philadephia	822,340	6.88
D	Cleveland	661,278	5.53
E	Richmond	948,516	7.94
F	Atlanta	1,565,732	13.10
G	Chicago	1,207,448	10.10
H	St. Louis	472,930	3.96
I	Minneapolis	360,194	3.01
J	Kansas City	713,393	5.97
K	Dallas	869,866	7.28
L	San Francisco	2,203,063	18.44

We restrict the analysis to the lower 48 states and the District of Columbia (thus excluding Hawaii and Alaska) and consider only legs with origin and destination locations in these states, reducing the original dataset to $11,759,420$ bills (98.40% of the original data) and $14,376,232$ trajectory legs (98.38%).

The spatial resolution of the dataset is given by 41, 106 zip codes, with mean linear extent of 14 km. The mean linear extent of the lower 48 states is 2, 842 km defining the bounds of the system. For each zip code Z_i we use centroid information to associate with each report a longitude/latitude location $\mathbf{x} = (\Theta, \varphi)$, such that each trajectory n corresponds to a sequence of geographic locations $\mathbf{X}_i$ with $i = 1, \ldots, L_n$:

$$t_n : \quad \{\mathbf{X}_{n,0}, \Delta T_{n,1}, \mathbf{X}_{n,1}, \ldots, \Delta T_{n,L_n}, \mathbf{X}_{n,L_n}\} \quad \text{with} \quad n = 1, \ldots, N_0 \tag{1}$$

where $\mathbf{X}_{n,0}$ is the initial entry location, and $\Delta T_{n,i} = T_{n,i} - T_{n,i-1}$ are inter-report times.

1.1. Geographical distributions

Based on these trajectories we define the density of initial entries as

$$p_{IE}(\mathbf{x}) = \frac{1}{N} \sum_{n=1}^{N} \delta(\mathbf{x} - \mathbf{X}_{n,0}) \tag{2}$$

and of reports as

$$p_R(\mathbf{x}) = \frac{1}{N} \sum_{n=1}^{N} \frac{1}{L_n} \sum_{i=1}^{L_n} \delta(\mathbf{x} - \mathbf{X}_{n,i}). \tag{3}$$

In order to assess the spatial distribution of reports and initial entries and to quantify the correlation with the population density we compute the number of reports and initial entries for each of the $M = 3,109$ counties in the lower 48 states. Defining for each county k a characteristic function

$$\chi_k(\mathbf{x}) = \begin{cases} 1 & \text{if} \quad \mathbf{x} \in P_k \\ 0 & \text{otherwise} \end{cases} \tag{4}$$

where P_k is the polygon defining the county's interior, the number of reports and initial entries in county k are given by

$$m_R(k) = \langle \chi_k \rangle_R = \int \chi_k(\mathbf{x})\, p_R(\mathbf{x})\, \mathrm{d}\mathbf{x} \quad \text{and} \quad m_{IE}(k) = \langle \chi_k \rangle_{IE} = \int \chi_k(\mathbf{x})\, p_{IE}(\mathbf{x})\, \mathrm{d}\mathbf{x},$$

respectively. Figure S1 compares the distribution of reports $m_R(k)$, initial entries $m_{IE}(k)$ and the population $P(k)$ of the 3, 109 counties. As all three quantities are positive and vary over many orders of magnitude, the maps depict $\log_{10}(m_R)$, $\log_{10}(m_{IE})$ and $\log_{10}(P)$. Qualitatively, reports and initial entries correlate strongly with the population density. Computing the correlation coefficient of the logarithmic quantities yields $c(R, P) = 0.933$ and $c(IE, P) = 0.819$. Despite the expected increase of $m_R(k)$ and $m_{IE}(k)$ with $P(k)$, only the report count increases approximately linearly with population size, whereas initial entries show a deviation for small populations. We believe that this deviation is a consequence of the social difference between the subpopulation of "Georgers" that are responsible for initiating bills and entering them into the system, "actively" playing the game, and the larger group of people that randomly receive a bill and report it, "passively" participating. This hypothesis could explain that areas with higher population densities contain a larger proportion of internet-savvy communities that are inclined to become Georgers and initiate bills. In order to exclude a potential bias caused by this effect we exclude all the legs in (5) that contain an initial entry as the origin, i.e. we only investigate the reduced set

$$t_{2,n} : \quad \{\mathbf{X}_{n,1}, \Delta T_{n,2}, \mathbf{X}_{n,2}, \ldots, \Delta T_{n,L_n}, \mathbf{X}_{n,L_n}\} \quad \text{with} \quad n = 1, \ldots, N_0 \tag{5}$$

that excludes the first legs of all t_n. Excluding the first leg reduces the number of bills to 4, 743, 330. However, the key results, e.g. the border structures discussed in the main text, are robust against the inclusion of initial entries. Computing mobility networks based on either set, t_n or $t_{2,n}$ does not change the observed pattern significantly.

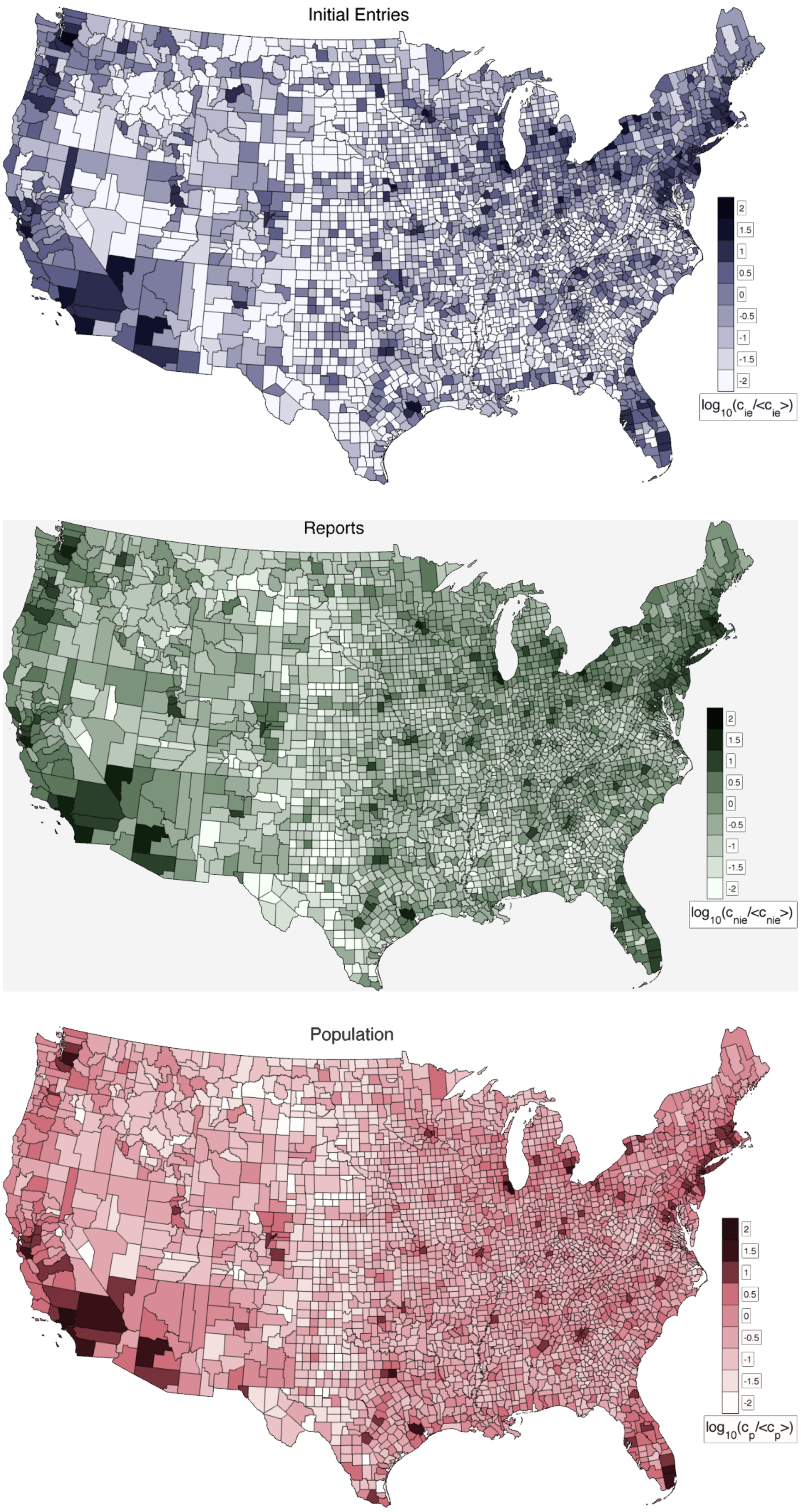

Figure S1: The frequencies of reports (top) and initial entries (middle) correlate with the county population (bottom) in the lower 48 states.

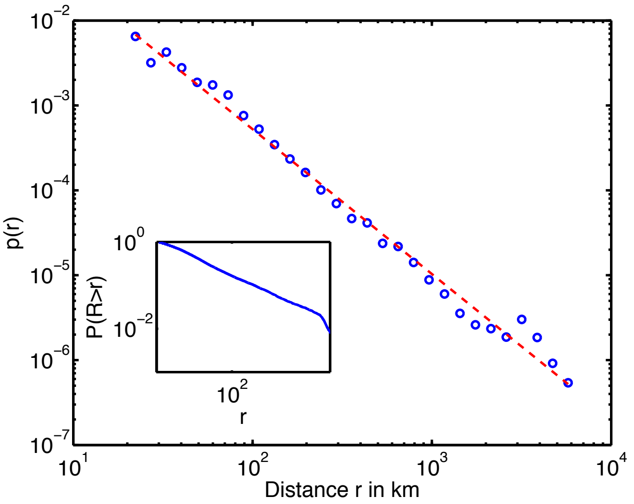

Figure S2: The estimated probability $p(r|t < \tau)$ of a bill traversing a distance r in time $t < \tau$ where $\tau = 4$ days. In red a maximum likelihood fit of the the function $p(r) \sim r^{-(1+\beta)}$ with $\beta = 0.7056$.

1.2. Distance and time: spatially averaged quantities

From $t_{2,n}$ we extract pairs of spatio-temporal leg distances $\{d_s(\mathbf{X}_{n,i}, \mathbf{X}_{n,i-1}), \Delta T_{n,i}\}$, where $d_s(\cdot, \cdot)$ denotes the distance on a sphere (shorter segment of the great circle that passes through both points). This type of dataset was first investigated in 2006 based on a much smaller core dataset of bill trajectories [4]. In particular, the combined probability density (pdf)

$$p(r, t) = \langle \delta\left(r - d_s(\mathbf{X}_{n,i}, \mathbf{X}_{n,i-1})\right) \delta\left(t - \Delta T_{n,i}\right) \rangle \tag{6}$$

was estimated as well as marginal pdfs $p(r)$ and $p(t)$. The central finding of the 2006 study was that $p(r) \sim r^{-(1+\beta)}$ and that the time evolution of the density (6) can be described by a bi-fractional diffusion equation. Here we reproduce some of the properties before we construct the mobility network used in the main text. Figure S2 shows the short time pdf of a bill traversing a distance r in a time $t < \tau$ where we chose $\tau = 4$ days. Using maximum likelihood we find this function can be described by a power-law

$$p(r) \sim \frac{1}{r^{1+\beta}} \quad \text{with} \quad \beta = 0.7056 \pm 0.0659.$$

This power-law describes the dispersal characteristics on a population averaged level. The short time distance pdf represents a dispersal kernel and for small times t approximates the instantaneous rate of traversing a distance r.

Complementary to this, temporal aspects of the process can be revealed by computing the pdf for the time t between reporting events given that these occur within a small radius $r > r_0$. Figure S3 depicts $p(t)$ for all legs with $r < 10\,\text{km}$ and a minimal inter-report time of $t_{\text{min}} = 1$ day. The inter-report times are described well by a power-law moderated by an exponential factor

$$p(t) \sim t^{-\alpha} e^{-t/T_0} \quad \text{with} \quad T_0 = 248 \pm 27, \quad \alpha = 0.99 \pm 0.05. \tag{7}$$

The observed power-law decay $\sim t^{-1}$ for times $t \ll T_0$ is intriguing. These type of decays have been observed in a multitude of contexts involving human acitvity, for instance the time between consecutive phone calls [10], emails [3] and the number of words between two identical words in texts [1]. A consequence of this law is bursting behaviour, i.e. given an event occured at time t_0 the probability rate that an event occurs immediately after the first is higher than expected from ordinary Poisson statistics. This behavior is best illustrated by the so-called hazard function $h(t)$

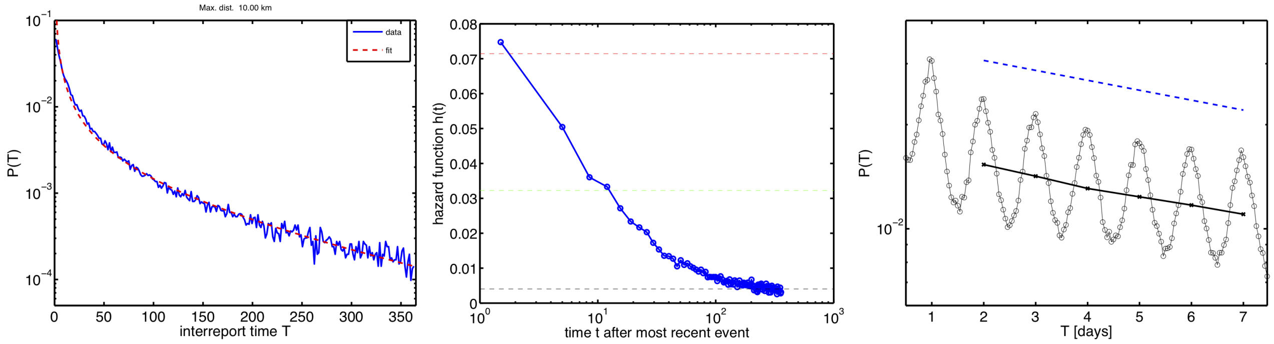

Figure S3: Inter-report time statistics. *Left:* the function $p(t|r < r_0)$ for $r_0 = 10\,\text{km}$. The observed function can be accounted for by an initial algebraic decay t^{-1} moderated by an exponential function for large arguments. The red dashed curved is a fit obtained maximizing likelihood. *Center:* The hazard function $h(t)$ that represent the instantaneous rate of an event at time t provided that an event occured at $t = 0$. The dashed lines represent reporting rates of once per 2 weeks (red), once per month (green) and once per $T_0 = 248$ days. *Right:* $p(t)$ for very short times. A zoom-in resolves daily oscillations modulated by the decay observed on the left. These oscillations indicate that users tend to report to the website at the same time of the day with the highest probability.

that quantifies the instantaneous probability rate of an event happening at t given that the last event occured at $t = 0$. If we let

$$P(\tau > t) = \int_t^\infty p(s)\,ds$$

be the cumulative probability that the second event occurs at a time τ later than t, the hazard function is defined by

$$P(\tau > t) = e^{-\int_0^t h(s)\,ds}.$$

For a Poisson process with rate γ we have

$$h(t) = \gamma \quad \Rightarrow \quad P(\tau > t) = e^{-\gamma t}.$$

The hazard function can be computed according to

$$h(t) = -\frac{\mathrm{d}}{\mathrm{d}t} \log\left[P(\tau > t)\right] = \frac{p(t)}{P(\tau > t)}.$$

Figure S3 depicts the function $h(t)$ for inter-report times in the WG data. For small times ($t < 1$ week) the probability rate for a report is of the order of one report per two weeks, which is also the expected time between two reports in this time window. For larger times ($t > 100$ days) the constant value of $1/T_0$ is approached, equivalent to one report in 3/4 of a year. Possible explanations of the bursting behaviour and the initial algebraic decay in $p(t)$ are a strong behavioral heterogeneity of players that participate in the game or an effective queueing in the system, i.e. bills may enter shops and intitially have a comparatively high likelyhood of leaving, being “on top of the stack”. As time passes these bills may “get stuck” and equilibrate to the long time scale present in the system.

1.3. Definition of the mobility network

From the trajectories defined by (5) and the characteristic functions of the counties (4) we construct a matrix $\tilde{w}_{ij}$ that counts the number of legs which originate at county i and terminate at j,

$$\tilde{w}_{ij} = \sum_{n=1}^{N} \sum_{k=2}^{L_n} \chi_i\left(\mathbf{X}_{n,k-1}\right)\, \chi_j\left(\mathbf{X}_{n,k}\right)\, \Theta(T - \Delta T_{n,k})$$

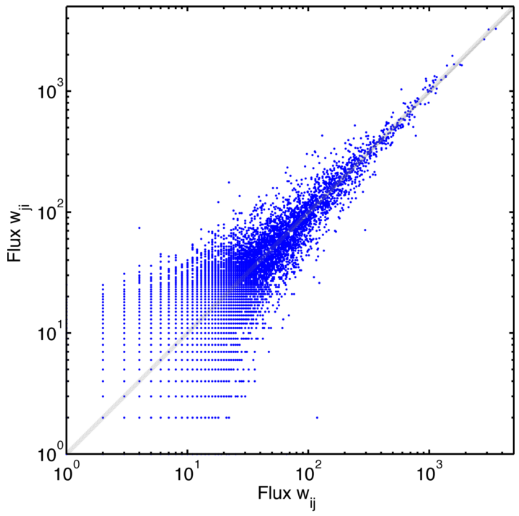

Figure S4: Symmetry of flux network $\tilde{w}_{ij}$.

where $\Theta(\cdot)$ is the Heaviside step-function. In order to exclude potential biases induced by initial entries we ignore the first leg of all trajectories ($k = 2$ in the above sum). This choice is motivated by the fact that the community of individuals that initiate bills might be less representative than those that find bills and report them. Indications that this might have an effect are supported by the different scaling behavior of initial entry frequencies with population as compared to report frequencies with population. The factor $\Theta(T - \Delta T_{n,k})$ excludes legs that have an inter-event time larger than time T. The matrix $\tilde{w}_{ij}$ need not to be symmetric, as the flux of bills from $i \to j$ need not equal those that travel $j \to i$. However, as Fig. S4 indicates the flux matrix is statistically symmetric. Plotting $\tilde{w}_{ij}$ agains $\tilde{w}_{ji}$ indicates a clear mean linear relationship. Since we base our analysis on the flux of money between two given counties we symmetrize the network and use w_{ij} in our analysis defined by

$$w_{ij} = \frac{1}{2} (\tilde{w}_{ij} + \tilde{w}_{ji})$$

which of course also depends on the time threshold parameter T. Choosing the optimal value for T is a trade-off between trying to estimate instantaneous flux, i.e. choosing T as small as possible, and using as many legs as possible to decrease fluctuations, i.e. choosing large values for T. Choosing a value for $T < 30$ days for instance rules out bills that visit a Federal Reserve Bank in between reports in counties i and j, as bills that enter FRBs do dot return to circulation until approx. 3–4 weeks after entering the FRB. As shown in Sec. 2, the probability of having an interfering visit to an FRB remains very small even if T is increased to a few months. To make sure that our results do not significantly change as the parameter T is varied we performed the analysis for various values of T ranging from a few days to $T = 1$ year. The computed border structure does not significantly depend on the value of T. Decreasing T thins out the network and reduces the overall connectivity, yet the effects are similar to bootstrapping the network randomly, a process that also does not change our results and is discussed in Sec. 3.3.

2. Validity of currency flux as a proxy for human mobility

All US currency is manufactured in Washington, D.C. or Fort Worth, TX by the Bureau of Engraving and Printing for the Department of the Treasury. Every bill is stamped as belonging to one of the 12 Federal Reserve banks, although in practice this only means that a bill originates from one of these banks the first time it's put into circulation. The Fed banks then sell currency to commercial banks, and from there currency enters general circulation. A bill may circulate between banks, individuals, and other businesses for some time before being returned to the Fed. When a bill is brought back to one of the Feds, its fitness is evaluated (at whichever branch of the Fed receives the bill; currency is not necessarily returned to the branch of origin to be evaluated). If it is unfit the bill is destroyed, and if it is fit, the bill is placed into a first in, first out queue. As regional banks require currency, the Fed draws bills out of the queue to fill this demand and distributes them back into circulation.

The cash services provided by the FRB system are used heavily by commercial banks: every day, the Federal Reserve Bank of New York alone processes some 20 million bills and destroys about a quarter of them.[1] The FRB reports that approximately 35% of all currency deposited in the system is destroyed,[2] and the Bureau of Engraving and Printing produces about 38 million new bills every day to satisfy demand for currency.[3] This large amount of currency moving through the arms of the FRB might have a strong effect on the geographic circulation of dollar bills. However, the number of bills in circulation is measured in the tens of billions (cf. Table S3), and in this section we demonstrate that in spite of the volume of currency processed by the Fed, measuring events such as this in our database is extremely unlikely.

Denomination ($)	1	5	10	20	50	100
Number of bills (billions)	7.7	1.8	1.5	4.9	1.1	3.8

Table S3: Amount of bills in circulation December 2000. `http://www.federalreserve.gov/paymentsystems/coin_currcircvolume.htm`

2.1. Time scale analysis

A general indication for why a bias from the Federal Reserve is unlikely to appear in our data comes from considering single dollar bills, which account for the largest portion of the dataset (see Table S1). A $1 bill typically lasts 1.8 years and visits the Fed twice in its lifetime, being destroyed the second time. This means that the average time between entries into the Fed is about 11 months, much longer than the typical inter-report time for $1 bills. Of course different denominations behave differently and this is explored in more detail below and later in Sec. 2.2, but here we describe how systematically applying time thresholds to our data can be used to reduce any possible bias introduced by the Fed.

We estimate the fraction of bill trajectory legs in our dataset that have not passed through the Federal Reserve. Each leg takes some time T, and the probability that the bill did not pass through the Fed in this time we call $p_0(T)$. Then the fraction of all legs that have not passed through the Fed is given by

$$f = \int_0^\infty p_0(T)\, p(T)\, dT$$

Here $p(T)$ is the probability density function of leg times; so the fraction of bills not passing through the Fed is the probability that one particular leg time T does not pass through, times the probability of observing this leg time, integrated over all possible leg times.

[1] `http://www.newyorkfed.org/aboutthefed/fedpoint/fed11.html`
[2] `http://www.frbservices.org/operations/currency/currency.html`
[3] `http://www.ustreas.gov/education/faq/currency/production.shtml`

Now we consider restricting the dataset to a time window τ; that is, we only consider legs with $T \leq \tau$, and ask what fraction of these bills have not passed through the Fed. p_0 is as before, and we replace the complete pdf with a truncated pdf $p_\tau(T)$ and write $f(\tau) = \int_0^\infty p_0(T)\, p_\tau(T)\, dT$. The truncated pdf is calculated from the complete pdf:

$$p_\tau(T) = \begin{cases} p(T)/\int_0^\tau p(T)\, dT & T \leq \tau \\ 0 & \tau < T \end{cases}$$

Substituting this in we find

$$f(\tau) = \frac{\int_0^\tau p_0(T)\, p(T)\, dT}{\int_0^\tau p(T)\, dT}$$

The inter-report time distribution is obtained from our data set, and we develop a simple model for p_0.

A bill is either in the Fed or in circulation, and we can calculate the average time between entries into the Fed based on the average lifetime of a bill and the average number of times a bill visits the Fed before being destroyed. Let L be the average bill lifetime and N the number of times it returns to the Fed; then the average time between entries to the Fed is

$$T_- = \frac{L}{N-1}$$

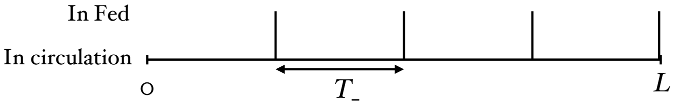

The denominator is due to the fact that the last time a bill enters the Fed, it is destroyed. Note that T_- is completely independent of the average time a bill spends in the Fed; only the average lifetime and average number of Fed entries matter. In general, we can say that the average time between Fed entries is the average time spent at the Fed plus average time spent in circulation, $T_- = T_f + T_c$.

As a worst-case estimate we begin by assuming that Fed events take very little time, $T_- \approx T_c$. Assuming that entering the Fed is a Poisson process, then given an inter-report time of T the probability that there was not a Fed event during this time is

$$p_0(T) = e^{-\gamma T}$$

where γ is the rate at which bills enter the Fed, given by $1/T_-$.

Alternatively, to estimate p_0 we could take into account the fact that the Fed does not process bills almost instantaneously. Rather it requires a considerable amount of time for a bill to be received by the Fed, processed, and recirculated back to a regional bank. Let T_f be the minimum amount of time a bill spends inside the Fed; then in this case we have

$$p_0(T) = \begin{cases} 1 & T \leq T_f \\ e^{-\gamma(T-T_f)} & T_f < T \end{cases}$$

The amount of time a bill may get “stuck” at the Fed varies according to the time of the year, the denomination of the bill, and other factors. However, bills are never recirculated within three weeks of initially being received.

These two possible models for p_0 lead to different estimates of $f(\tau)$, both parametrized on the average lifetime of a bill and average number of Fed entries. These parameters vary substantially over the different denominations, and here we present a detailed investigation for \$1 bills and \$20 bills,

Table S4: Average lifetimes of the various denominations (`http://www.federalreserve.gov/generalinfo/faq/faqcur.htm`)

Denomination ($)	1	5	10	20	50	100
Average Lifetime (days)	630	480	540	720	1650	2670

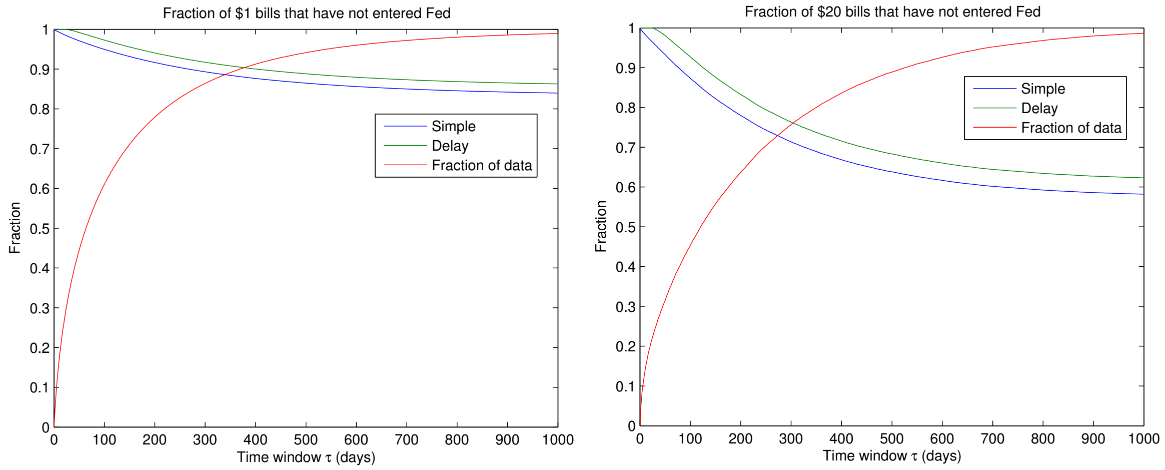

Figure S5: Fraction of trajectories that have not passed through the Fed and fraction of trajectories that remain given a time window τ. For $1 bills we use $L = 630, N = 2$ (e.g. approximately half of the $1 bills entering the Chicago Fed are destroyed), and for $20 bills we use $L = 720, N = 4$ (cf. Tab. S4). For both models with delay we use $T_f = 21$. (This number is conservative; it could be much higher, which would move the green curve further right.)

as they account for the largest portion of our dataset and the largest portion of bills in circulation. In Figure S5 the blue lines indicated the fraction of bills that have not passed through the Fed using the simple model with no sticking time, the green lines are the model with a sticking time, and the red lines indicate the fraction of all of the trajectories in our dataset that remain after thresholding at the given time window. The graphs indicate that, for example, 80% of $20 bill trajectories take less than one year ($\tau = 365$), and of those, about 70% have not passed through the Fed. For the $1 bills the situation is even more favorable. After a year, 90% of trajectories remain, and 88% of those have not passed through the Fed.

Although the graphs in Figure S5 indicate that, in general, a very high proportion of our dataset is unaffected by the Fed, they also make it clear that, as we reduce the time window, we will systematically filter out those legs that are affected by the Fed. We therefore plot the correlation between link weights in the full, time-unrestricted network and various time-thresholded networks, where links are constructed using only bill trajectories with $T \leq \tau$. Figure S6 (left) shows that, although decreasing the time window certainly affects the strength of links in the Where's George network, it does this in a predictable way. If the actions of the Fed disproportionately increased the strength of links in and around the reserve districts, then these links should be disproportionately weakened by reducing the time window. However, we do not observe this effect; on the contrary, reducing the time window reduces the strength of all links in the network proportionately. Note that the blue line in Figure S6 (left) indicate a time window inside of which it is impossible for the Fed to introduce any bias whatsoever into the link weights, and yet here the link weights are proportional to the weights of the unrestricted network as well.

An alternative, albeit unlikely interpretation of these results is that for every pair of counties the flux of currency due to relocation by banks is exactly proportional to flux of currency due to human

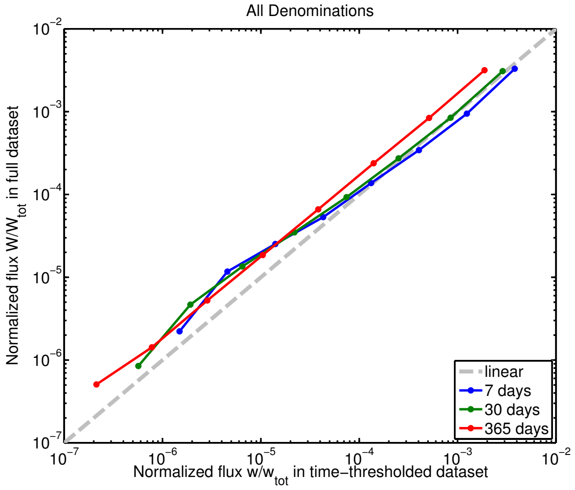

Figure S6: Comparing the unrestricted network to various time-thresholded networks, for all denominations combined. The nearly linear relationship on a doubly-logarithmic scale indicates that the time-thresholded networks are proportional to the unrestricted network.

mobility, that is

$$w_{ij} = w_{ij}^{\text{humans}} + w_{ij}^{\text{banks}} = (1 + A)\, w_{ij}^{\text{humans}}.$$

This would have no effect on the results presented in this paper, since both methods used here are insensitive to scaling.

Although the Federal Reserve System is certainly responsible for processing a tremendous number of bills and undoubtedly affects the geographic circulation of money, the timescales involved generally rule out the possibility that this biases our observations. We constructed a model based on reasonable assumptions that gives a lower bound for the fraction of trajectories we observe that are affected by the Fed, and find that this already shows that there should not be a strong bias. Moreover, we find by correlation of the full network with time thresholded networks that the actual bias present in our data is far less than what the worst-case model predicts.

2.2. Denomination analysis

Furthermore, we check the behavior of different denominations. The distribution of various denominations is listed in Table S1. The vast majority of bills are singles, yet a small but significant number of bills in the WG dataset are of higher denomination. The exception is the $2 bill that is rare and plays a more dominant role as a collectors item than as a means for cash payments. As various denominations serve a different purpose in circulation we expect to see differences in the statistical properties. The figure below is a schematic showing the important features of currency circulation.

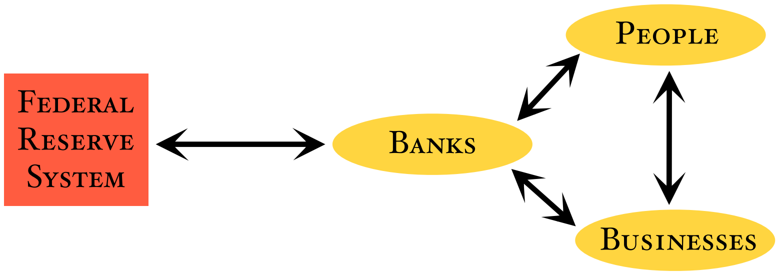

Consider how the different denominations of currency are typically used: larger denominations are used by people to purchase items from businesses. A customer enters, makes a purchase with some amount of currency, and receives smaller-denomination bills in return (along with a good or service). This common scenario, repeated many times, means that businesses have a large demand for low-denomination bills that is not satisfied by customers, only by banks, and that people have a demand for high-denomination currency that is generally satisfied by banks, not businesses. This means that, for example, $20 bills are likely to move through this diagram in a generally clockwise direction, passing from banks to individuals to businesses and back to banks, while $1 bills likely pass back and forth between people and businesses many times before returning to a bank. Furthermore, the more frequently a bill returns to a bank, the more likely it is sent to the Fed, and the more frequently a bill is given to an individual, the more likely the bill is reported on wheresgeorge.com.

Analysing the statistics for each denomination can reveal to what extent business-to-business transport of money impacts on the spatial relocation of currency and to what extent this generates the flux matrix w_{ij} (instead of humans carrying currency from place to place, which is a key assumption of our approach). If the data indicate that higher denominations exhibit a higher probability of entering banks or, equivalently, spend less time in peoples' hands, it is reasonable to assume that this difference in local circulation patterns would also show a trace in the spatial patterns, i.e. the statistics of distances traveled.

In order to investigate differences and similarities in local circulation patterns we compute the inter-report time statistics for each denomination (except $2 bills); the results are depicted in Fig. S7. We compute the mean and variance of inter-report times as well as the cumulative probability $P(\Delta T > t|\$)$ that an inter-report time ΔT is larger than a given time t. The results indicate that smaller denominations do indeed not only exhibit identical properties in their subgroup but they also differ significantly from those of the larger denominations.

Note that the difference in $p(\Delta T > t|\$)$ cannot be attributed to the smaller size of samples in the higher denominations. For example the number of $10 bills and $20 bills are approximately the same, nevertheless $p(\Delta T > t|\$)$ is quite different for these two denominations. We believe that this temporal difference in smaller denominations on one hand and large denominations on the other is caused by the different local paths of circulation these bills take. Larger bills have a much smaller likelihood of crossing the counter in both directions and tend to take a more circular path from banks

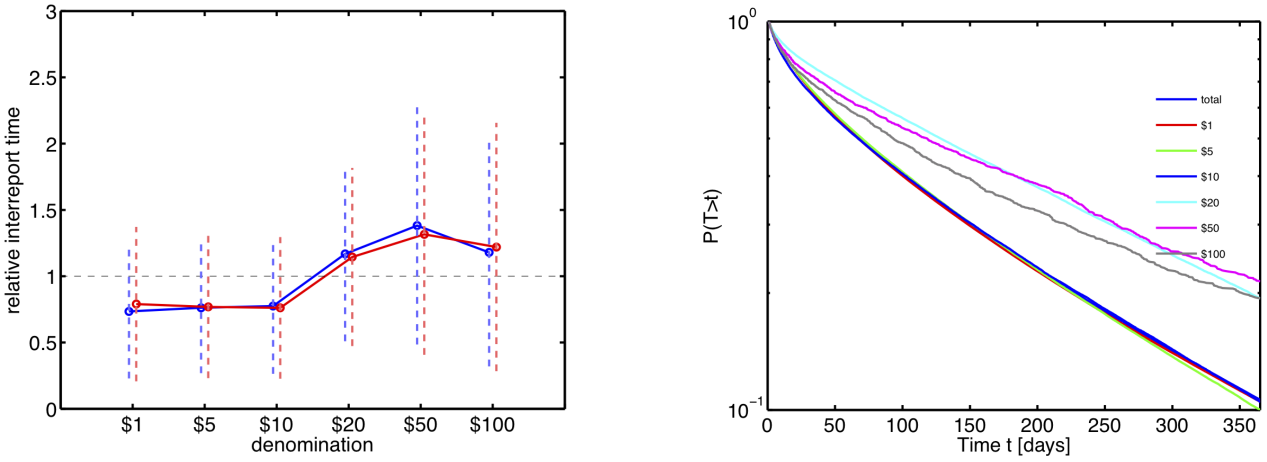

Figure S7: Inter-report time statistics for the different denominations in the dataset. *Left:* The relative mean inter-report time. The small denominations \$1, \$5 and \$10 exhibit approx. the same mean inter-report time that is also significantly smaller than that of the higher denominations. *Right:* The cdf $P(\Delta T > t|\$)$ for each denomination. We observe again a power-law moderated by an exponential for large t. Interestingly, the smaller denominations exibit almost identical pdfs whereas higher denominations deviate.

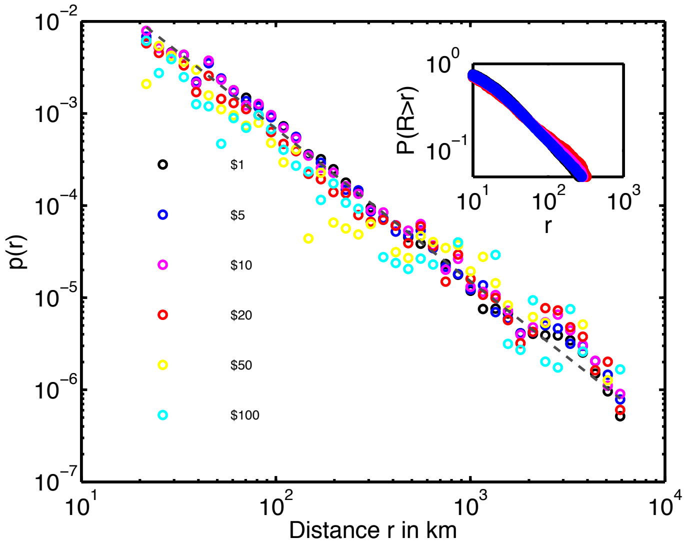

Figure S8: The short time pdf $p(r|\$)$ for all denominations except the \$2 bills. All denominations exhibit a power-law decay with an exponent consistent with that of the entire population of bills.

to people to shops to banks etc. If this is the case, and business-to-business relocation of currency plays a more dominant role in the spatial dispersal of currency, then we expect to see a different behavior in spatial statistics of small denominations on one hand and large denominations on the other.

Figure S8 depicts the short time distance pdf $p(r|\$)$ for all denominations. Despite significant differences in the distribution of inter-report times in local circulations, all denominations exhibit similar functional shapes of $p(r|\$)$. This result speaks against the hypothesis that geographic relocation is dominated by business-to-business transportation of currency and we believe that the universal shape of $p(r|\$)$ is yet another reason to assume that the dominant factor in the flux of currency is human mobility.

3. Ensembles of high-modularity partitions

3.1. Finding optimal partitions

Our method relies on finding several different high-modularity partitions, which restricts the range of applicable algorithms. For example, the deterministic divisive algorithms described by Newman and Girvan [14] cannot find several different local maxima of the modularity function. In contrast, Monte Carlo algorithms return different partitions with probabilities that monotonically increase with the corresponding modularity values, one of which is the simulated annealing algorithm described by Guimerà and Amaral [12]. Additionally, this algorithm has been found to be the best-performing in terms of correctly identifying modules in networks with artificial community structure in a survey by Danon et al. [5], which lead us to choose this algorithm for our work.

The partition vector P is initialized such that each of the N nodes is in its own module, $P_i = i$. Alternatively, one could randomly assign each node to one of a few modules to form the initial partition. We found, however, that in this case the algorithm will split these few large modules into a large number of very small modules before slowly merging them into the final result. Since splits of large modules, involving a recursive simulated annealing run, are computationally very expensive, we avoid them by starting with a partition of single-node modules.

A small modification of the partition is then made (see below) to obtain a new partition P' and its effect on the modularity value, $\Delta Q = Q(P') - Q(P)$. If $\Delta Q > 0$, the new partition is better than the old one and we replace $P = P'$. If $\Delta Q < 0$, the partition is only accepted with probability $p_T(\Delta Q) = \exp(\Delta Q/T)$, where T is a "temperature" that controls the typical penalty on Q we are willing to accept with the new partition P'.

This procedure is repeated a number of times, initially with a high $T = T_0$ accepting modifications with large negative impact on modularity and therefore allowing to sample multiple local maxima. After $\mathcal{O}(N^2)$ modifications, the temperature is lowered by a *cooling factor* c. When T is small enough, worse partitions are not accepted anymore and the partition P has "annealed" into a local maxima of the modularity landscape $Q(\cdot)$.

During each temperature step, we intersperse $f\,N^2$ local with $f\,N$ global modifications of the partitions, f being a parameter. A local modification is a switch of one node to another, randomly selected, module, while a global modification can be a merge of two or a split of one randomly selected module. Finding a suitable split of a module that is not immediately rejected is done by recursively running a simplified version of the simulated annealing algorithm on it: The module in question is extracted and treated as an independent network, initially randomly partitioned into two modules. Only local modifications are allowed while annealing this bipartition into a local modularity maximum. Afterwards, the split module is replaced into the full network and evaluated against the modularity value of the full partition.

We observed that the global structure of the partition is found quickly by the algorithm and mostly only local modifications are accepted at low temperatures. Since the split operations are computationally intensive, we therefore track the number of rejected split modifications in each temperature step and reduce the probability of future trials if that number is high.

To generate the large ensemble of partitions, we used $T_0 = 2.5 \cdot 10^{-4}$ as initial temperature, $c = 0.75$ as the cooling factor, and $f = 0.05$. We abort the procedure and accept the partition as "optimal" if no better partition is found in three consecutive temperature steps. The parameters are less conservative than those proposed in [12], but we found the algorithm to perform very well and in acceptable runtime with this configuration.

3.2. Superposition of partitions and limitations of modularity

As described in the main text, the key feature of our method is the ability to extract statistically significant properties of an ensemble of partitions, instead of focusing on a single realization. This is

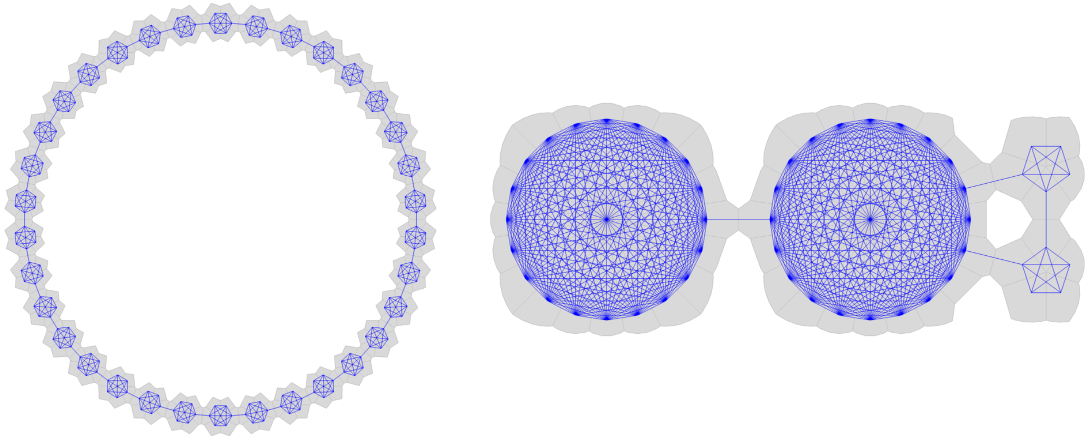

Figure S9: Two networks that expose the resolution limit problem with modularity [9]. The shaded areas indicate an artificial geography for nicer visualization of the boundaries in the next figures. *Left:* A ring of 34 cliques, each of 6 nodes and connected to their neighbors by single links. *Right:* A network of two 20-node cliques and two five-node cliques.

motivated by the finding that many partitions may yield similarly high modularity values, yet differ significantly in structure. In fact, it is straightforward to construct networks of which several distinct partitions with equal modularity value exist. This *degeneracy of modularity* was independently found by Good et al. [11] and marked as a drawback of the modularity measure.

Our proposed method combines an ensemble of partitions by focusing on the boundaries of a partition ("Which adjacent nodes are separated into different modules?") rather than its volumes ("Which nodes are grouped together?"), and then computing for each boundary the fraction of partitions in which it exists. Because we are interested in geographically embedded networks and modules are virtually always spatially compact in our case, we can restrict ourselves to boundaries that are also real geographical borders between nodes. However, the idea can be easily generalized to non-geographical networks, at the expense of convenient straight-forward visualization. Since all partitions in the ensemble have a high modularity value, this method highlights similarities and differences in degenerated partitions, yielding a unique "partition" (or to be more precise, a map) of the network and thus overcoming the degeneracy problem.

Another drawback in the concept of modularity is the so-called *resolution limit*, as reported by Fortunato and Barthélemy [9]. The authors present two artificial, unweighted networks that exhibit an intuitively very clear community structure, yet partitions exist that do not reflect this structure but have a higher modularity value than the partition that does. In particular, these networks are constructed by connecting multiple fully connected graphs ("cliques") with single links (cf. Fig. S9). It is clear that every clique should be grouped into one module, but the best partition according to modularity will group multiple cliques together. This only occurs if the cliques are small (in terms of number of links) compared to the full network, thus the modularity measure cannot detect communities below a certain resolution limit.

In our method every single partition obviously suffers from this limitation as well. However, the resolution limit can be alleviated by looking at an ensemble, if enough small modules exist to create degeneracies. To illustrate this, we applied our method to the two example networks from Ref. [9]. Figure S9(left) shows a ring of 34 6-cliques, all connected to their neighbors by a single link. The intuitive partition in which each clique is in its own module has modularity $Q_{\text{real}} = 0.9081$ while a partition that groups pairs of cliques together has $Q_{\text{opt}} = 0.9099$. However, two distinct partitions exist that group pairs of cliques. Thus, an ensemble of optimal partitions will be composed out of those two partitions, yielding a boundary map in which every boundary between two cliques appears in 50% of the ensemble partitions. For nicer visualization, we created an artificial geography for

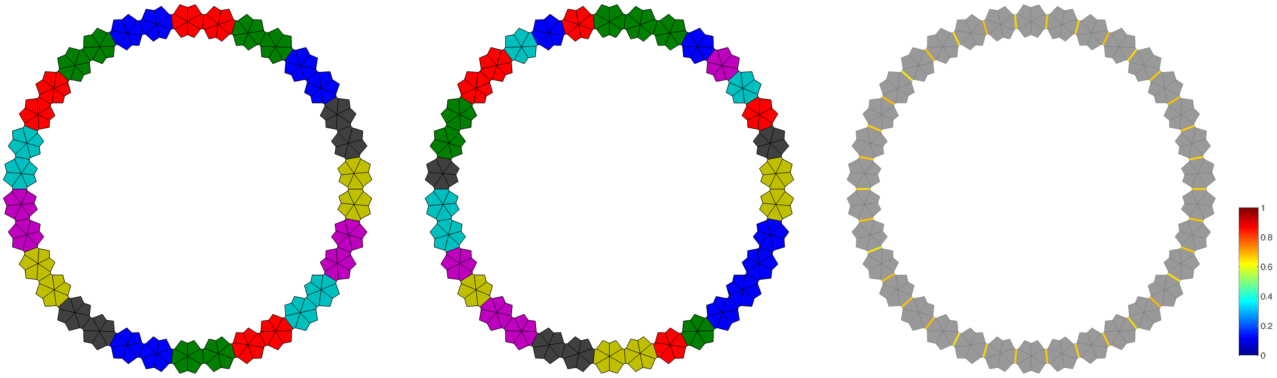

Figure S10: *Left:* The optimal partition in the clique ring groups pairs of cliques together (the same color is used for multiple modules). *Center:* Example of a partition found by the modularity optimization algorithm. *Right:* Boundaries in the clique ring are found between every clique, not just every other. Color codes the fraction of partitions in which the boundary was found.

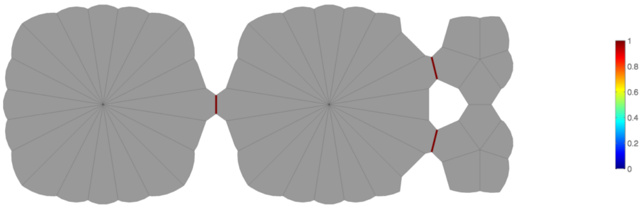

Figure S11: Boundaries found in the clique network shown in Fig. S9(right). Our algorithm is not able to find a boundary between the two small cliques.

this network and computed partitions and boundaries, shown in Fig. S10. Due to the nature of our algorithm, the resulting partitions contain a few n-tuples of cliques and single-clique modules that have not been split or merged into perfect clique-pairs before the termination criterion, and thus the observed boundaries are stronger than expected.

The second network proposed in Ref. [9] is constructed from two 20-cliques and two 5-cliques (cf. Fig. S9, right). Here, the two smaller cliques are merged into one module by the optimal partition ($Q_{\mathrm{opt}} = 0.5426$), although one would again expect each of them to be in its own module ($Q_{\mathrm{real}} = 0.5416$). Our method is not able to capture the intuitive community structure in this case (cf. Fig. S11), because no degeneracy exists (the partitions in which only one of the small cliques are grouped with the large one, but not the other, are too far from the optimum to be produced by the algorithm, $Q_{deg} = 0.4959$).

If we extend the network such that four small cliques exist, the partition which groups all cliques into their own modules is still suboptimal to any partition that groups together more than one of the

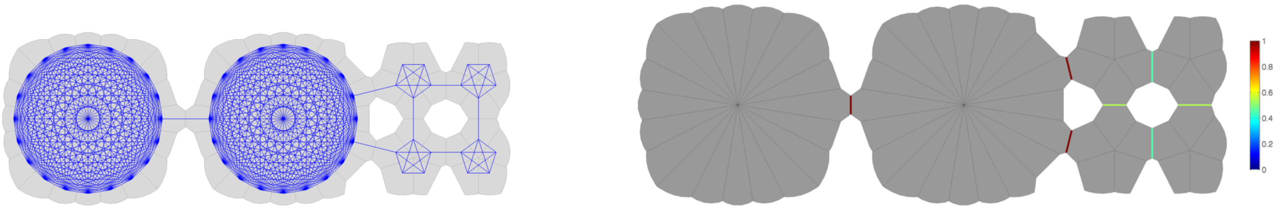

Figure S12: Modification of the clique network in Fig. S9(right). Because there are multiple high-modularity partitions that group the smaller cliques into pairs, our method can detect the correct community structure in this case.

small cliques, but degeneracies are created and the ensemble of partitions reveals the true community structure in this network (cf. Fig. S12).

In conclusion, our method is able to dissolve both the degeneracy and resolution limit problems if enough small modules exist to create degeneracies. In fact, we observe small "building blocks" in the WG data that are not seen in single partitions but emerge from the superposition of a partition ensemble.

3.3. Bootstrapping the Where's George data

In order to test the robustness of our method against random data removal, we performed the following bootstrapping analysis. Starting with the full dollar bill dataset we have, and the resulting network weight matrix W with elements w_{ij}, we randomly remove single dollar bill reports until the total flux $f = \sum_{i,j} w_{ij}$ is reduced by a factor γ. Using this method we constructed several networks for $0 \leq \gamma \leq 0.95$ and computed an ensemble of 100 partitions for every value of γ, using the simulated annealing algorithm described in Sec. 3.1. We find that the modularity value is unaffected by bootstrapping even if 95% of the total flux is removed, although the number of modules in each partitions rises as the network is thinned out more than 85% (cf. Fig. S13). Also, the boundary structure emerging from superposition of all partitions is very robust under this procedure (cf. Fig. S14). At 20% of the original flux ($\gamma = 0.8$), approximately equal in volume to the long-range subnetwork discussed in the main text, virtually all of the boundaries found in the complete network are still identified, although the sparsity of the data evokes some singular counties. Even with only 5% of the flux, when boundaries become more fuzzy, some of the original structures are detected.

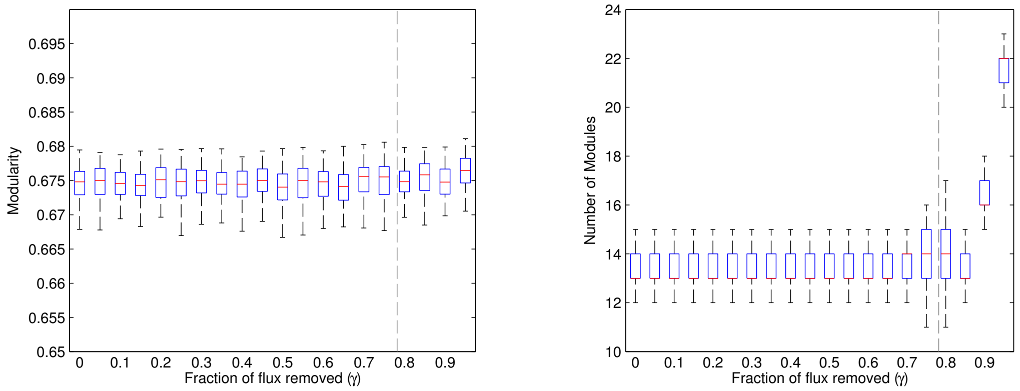

Figure S13: Distributions of modularity values and number of modules for an ensemble of 100 partitions computed for each value of the bootstrapping parameter γ. The dashed line corresponds to 78.2%, the amount of flux ignored by removing all links shorter than 400 km.

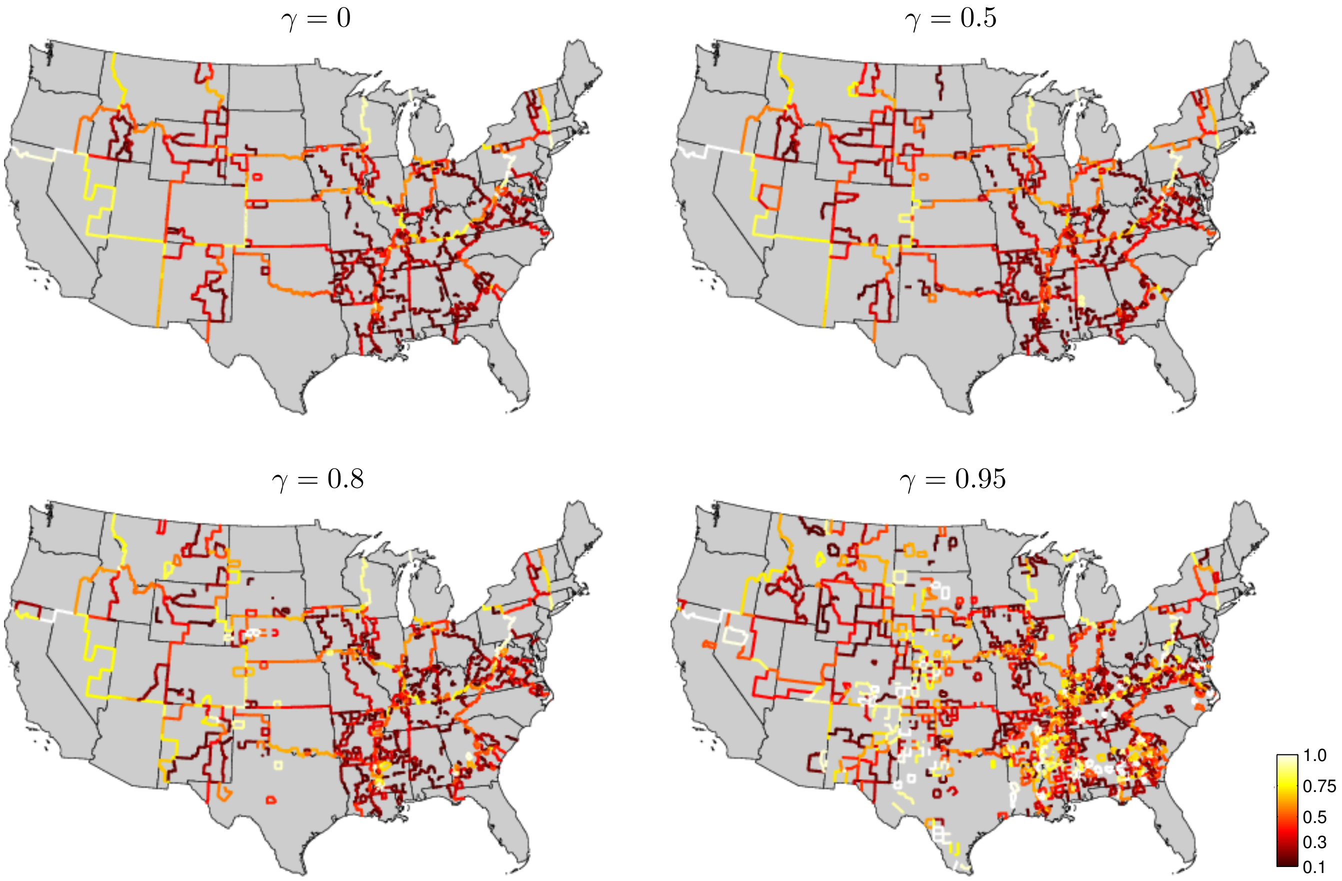

Figure S14: Linear superposition of 100 partitions for four different values of the bootstrapping parameter γ, color-coded according to the fraction of partitions they appear in.

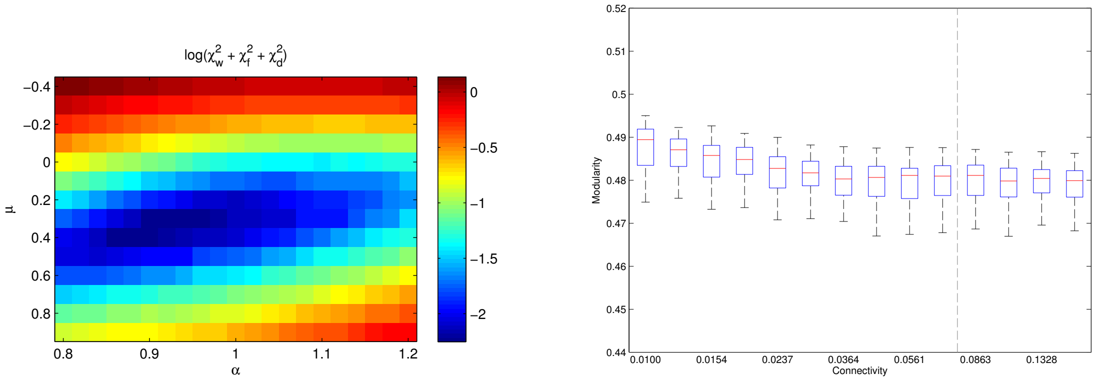

Figure S15: *Left:* χ^2 goodness-of-fit for different parameters of the gravity law. The minimum is at $(\alpha, \mu) = (0.96, 0.3)$. *Right:* Distributions of modularity values for an ensemble of 80 partitions each computed for snapshots of the model network at different connectivities. The dashed line corresponds to 0.0765, the connectivity of the real-data mobility network.

4. Gravity as a null model

This section provides details on how to we constructed the gravity model network that is referred to in the main text. In gravity models the interaction strength between a collection of sub-populations with geographic positions x_i, sizes N_i (obtained from census data[4]), and distances $d_{ij} = ||x_i - x_j||$ is given by

$$p_{ij} \propto \frac{N_i^\alpha \, N_j^\beta}{d_{ij}^{1+\mu}}$$

in which α, β and μ are the parameters.

To create a model network comparable to our data, we first compute p_{ij} for all counties i and j in the continental U.S. and normalize them such that $\sum_{i,j} p_{ij} = 1$. We then interpret these values as probabilities for a travel event to happen between the two counties (or, speaking in terms of the original data source, a dollar bill report). Thus, starting with all-zero link weights w_{ij}, we repeatedly draw a pair of nodes according to p_{ij} and increase the corresponding w_{ij} by one, until approximately the same connectivity (number of non-zero w_{ij}) as in the real-data network is reached.

We generated gravity networks for different parameter values and gauged them against our real data by comparing the distributions of first-order network statistics to find the best fit to our data. Distributions have been compared by log-binning the values and computing the χ^2 statistic

$$\chi^2 = \sum_i^n \frac{(N_i^G - N_i^R)^2}{N_i^R}$$

where n is the number of bins and N_i^G (N_i^R) is the number of values from the gravity (real-data) network in bin i.

Our real data is symmetric and node fluxes are proportional to population sizes, therefore we assume $\alpha = \beta \approx 1$ to narrow down the search volume in parameter space. We computed χ^2 for the distribution of link weights, node fluxes and geographical distances and used the sum of them, $\chi^2_w + \chi^2_f + \chi^2_d$, as the goodness-of-it measure. Figure S15(left) shows this quantity for $(\alpha, \mu) \in [0.8, 1.2] \times [-0.4, 0.9]$, from which we concluded that $\alpha = \beta = 0.96$ and $\mu = 0.3$ are the best parameter choices. The resulting network and first-order statistics are shown in Fig. S16.

[4] http://www.census.gov

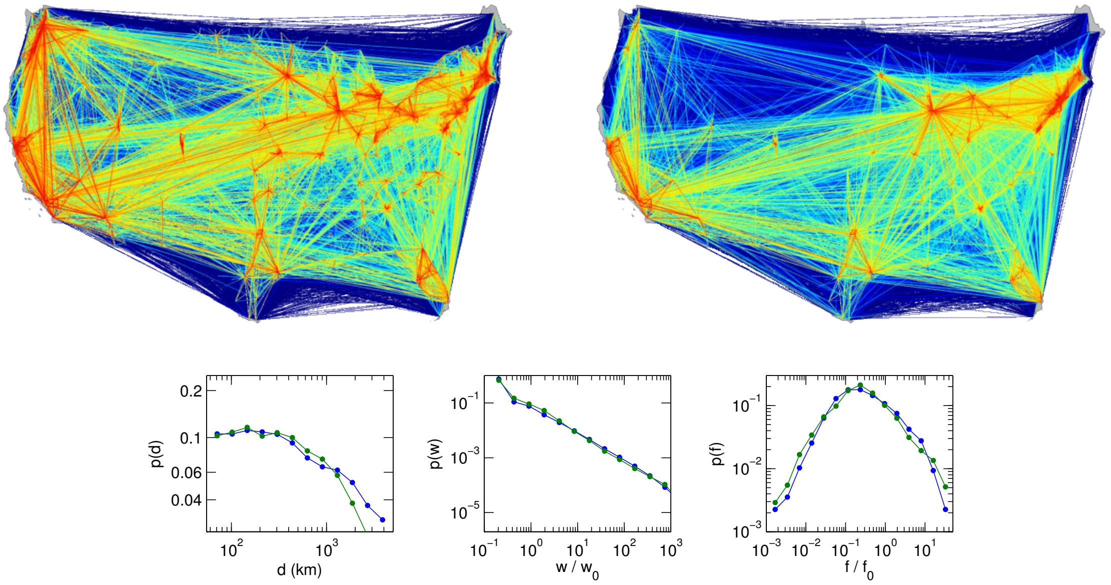

Figure S16: Comparison of the real-data network (top left) and the gravity model network with $\alpha = \beta = 0.96$ and $\mu = 0.3$ (top right). The bottom plot shows the distributions of geographical distances d, link weights w and node fluxes f in the real-data network (blue lines) and the gravity model (green lines).

Similar to the bootstrapping procedure described in Sec. 3.3, we tested the robustness of the community structure of the model network by generating snapshots of the network at different connectivities and computing an ensemble of 80 high-modularity partitions for each snapshot. We found that the modularity statistics are stable around the target connectivity of 0.0765 (cf. Fig. S15(right)).

5. Shortest-path tree clustering

5.1. Computing shortest-path trees

The shortest path from vertex i to vertex j is the series of edges that minimizes the total distance [6]. The distance along an edge for us is the inverse of the edge weight, as a highly-weighted edge indicates that two vertices are effectively proximal. (There are no edges with an infinite distance, because we do not define an edge between vertices if there is zero weight.)

The shortest-path tree rooted at vertex i is the union of all shortest paths between i and the rest of the vertices in the graph. We use the MATLAB interface[5] to the Boost Graph Library[6] to compute shortest-path trees. To prevent random fluctuations in our data from overwhelming the signal, we add a weak link between neighboring counties.

5.2. Measuring tree distance

A shortest-path tree can be easily represented as a vector of vertex labels $T = [t_k], k = 1 \dots N$, such that t_k is the label of the parent of vertex k, with a special symbol (perhaps 0) used to indicate the root. There are no disconnected nodes in the mobility network, thus each tree vector represents a single tree and not a forest. This representation lends itself to straightforward and meaningful comparisons between two trees.

We define two related measures of the dissimilarity between two trees. The first, called *parent dissimilarity*, asks the question, how many of the vertices in T_A do not have the same parent in T_B? We denote this by $z_p(T_A, T_B)$, and it is exactly the general Hamming distance of two symbol sequences, that is, the number of places where corresponding labels in T_A and T_B do not match. The second, called *overlap dissimilarity*, asks the question, how many edges do the two trees *not* share? It is defined as $z_o(T_A, T_B) = s_{max} - s(T_A, T_B)$. Here, s_{max} is the largest number of edges two trees could share, which is the number of vertices less one (since the root does not contribute an edge). $s(T_A, T_B)$ is the number of edges that T_A and T_B *do* share, and where z_p asks essentially the same question considering edges to be directed, z_o considers edges to be undirected. Also note that although we consider only the topology of trees when measuring their dissimilarity, the topology is determined by the weight of edges in the original graph and thus the mobility dynamics.

We compute both measures for each distinct pair of trees in our network and find that they are highly correlated (the Pearson correlation coefficient of the two sets is 0.9980). For this reason, and because of the more straightforward interpretation, we focus exclusively on z_p in the main text.

To test the stability of this measure we also added various amounts of noise to the original weight matrix; for example, adding 1% noise means that we adjusted each entry by a random number such that its perturbed value is within 1% of its original value. We then compute the set of shortest-path trees for the perturbed weight matrix, calculate the tree dissimilarities, and then compute the Pearson correlation of the original dissimilarities and the perturbed. The results (0.9995 for 0.1% noise, 0.9984 for 1% noise, 0.9937 for 5% noise) indicate the method is robust against small perturbations, and in addition we do not observe significant changes in the structure of borders determined by the perturbed matrices.

5.3. Hierarchical clustering

The measures described above produce a dissimilarity matrix well-suited for use with hierarchical clustering [8]. This technique iteratively groups data points together into clusters that are less and less similar; it begins by identifying the two points with the lowest dissimilarity and grouping them together, then finding the next-most-similar data point or group, and so on. When it is

[5] http://www.stanford.edu/~dgleich/programs/matlab_bgl/

[6] http://www.boost.org/doc/libs/1_41_0/libs/graph/doc/index.html

Linkage	$z_p(T_i, T_j)$	$1/w_{ij}$
single	0.6584	0.1883
average	0.8048	0.3757
complete	0.7197	0.1400

Table S5: Cophenetic correlation coefficients [15] for various linkage functions using parent dissimilarity of trees and inverse weight of links

necessary to compare the dissimilarity of one point (or group of points) with another group of points, a linkage function is used. There are several commonly-used linkage functions; we compute single linkages (comparing the shortest distance between two groups), average linkages (the average distance between two groups), and complete linkages (the greatest distance between two groups) and find that the average linkage produces the best fit to our data (cf. Table S5).

The result of the hierarchical clustering algorithm is a linkage structure that can be represented graphically with a dendrogram (Fig. S17). The radial lines in the dendrogram represent vertices in our network or groups of vertices, and the arcs represent a link that joins groups together in the hierarchy. The nearer an arc is to the center of the circle, the greater the dissimilarity between the groups joined by the arc.

Each arc corresponds to a geographic border between a set of counties, and the closer the arc is to the center of the circle, the more significant the border. At the outermost level, the dendrogram necessarily puts a border around each individual county, and we threshold at 30% of the height of the tree (corresponding to a dissimilarity $z_p = 41.6019$) for the analysis in the main text.

As you can see in Fig. S18, the groups identified by this procedure are spatially coherent, but may be divided into spatially disjoint regions at certain heights in the dendrogram.

Hierarchical clustering is also sometimes applied directly to the inverse of the weights, $1/w_{ij}$. We have investigated this method as well and find that it has several shortcomings. First, to apply a hierarchical clustering algorithm requires computing a dissimilarity for every pair of data points; since many pairs of counties are not directly connected by a link in our network (w_{ij} is zero), the inverse clearly does not exist and it is consequently necessary to add some noise to the weight matrix at the very first step, representing pairs of vertices that are 'extremely distant' but not disconnected. Second, the linkage structures produced from this approach fit the data poorly (cf. Table S5). Last, one can see by visual inspection of the dendrogram that this approach does not yield significant information. Comparing the dendrograms for the z_p and $1/w$ matrix, we see that in the shortest-path tree approach most of the links that appear higher in the tree (closer to the center) are linking together two groups that are strongly dissimilar from one another (seen by comparing the height of the parent link to the heights of the children links). In the inverse weight method, this is not true: links high in the tree are linking groups that are quite similar; that is, inverse weight clustering does not identify groups of strongly dissimilar vertices.

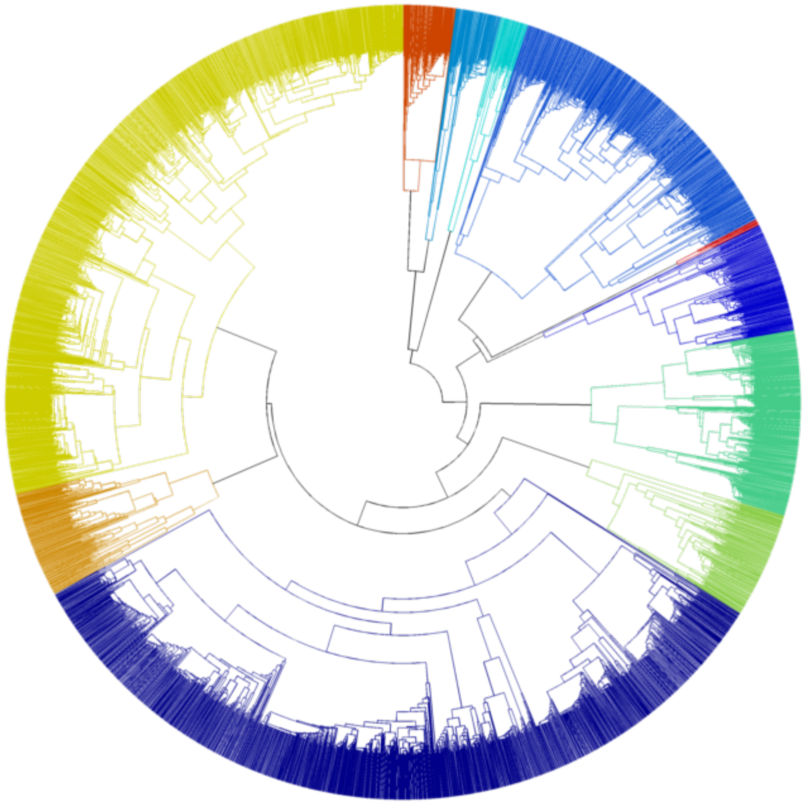

Figure S17: Hierarchical clustering with parent dissimilarity using average linkages; colors correspond to a particular partition depicted in Fig. S18

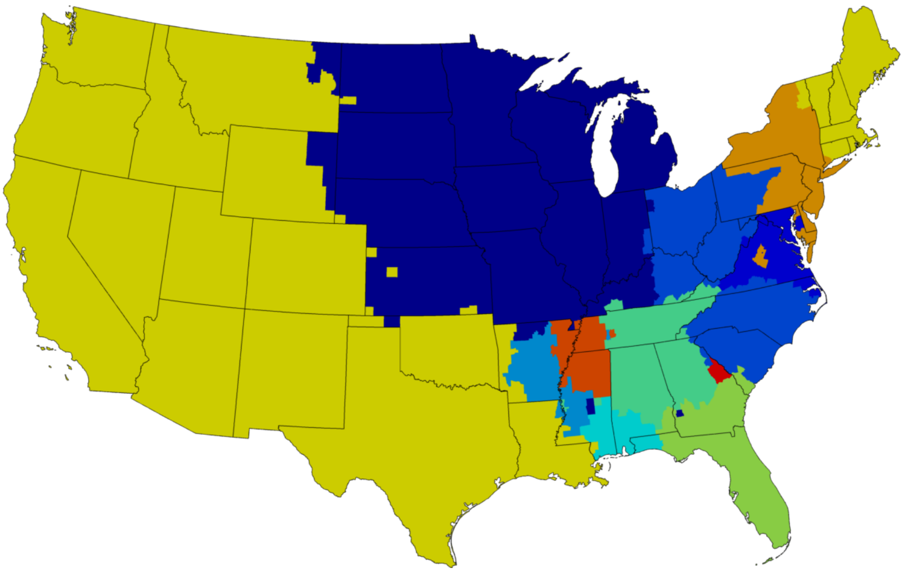

Figure S18: The geographic partition determined by cutting the dendrogram of Fig. S17 at a height of 95

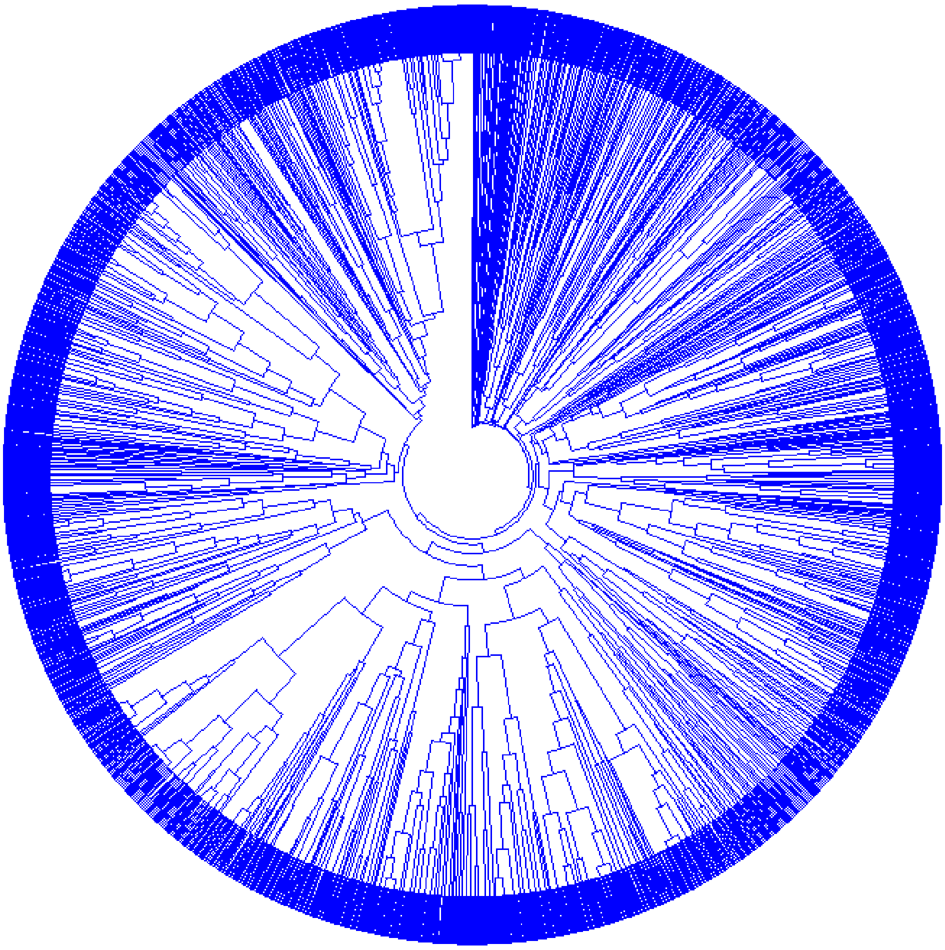

Figure S19: Hierarchical clustering of the inverse weight matrix with noise using average linkages

6. Significance and comparison of border structures

In this section, we describe how to compare boundary networks defined on a planar graph, in our case the county network of the continental US excluding Alaska. A *boundary network* b is simply given by assigning a nonnegative number w to each edge between adjacent counties: If the two counties are not divided by b then $w = 0$. Otherwise $w > 0$ implies that the border shared between the two counties has the strength w. In the main text, we described how to generate such a boundary network by superposition of many partitions of the Where's George money travel network, see Figure 2 in the main text. We denote this boundary network by the modularity boundaries b_M.

6.1. Measuring overlap of two boundary networks

We want to quantify how much information the modularity boundaries b_M shares with e.g. a state network, a random network or a boundary network generated with another method.

For this we essentially need to determine the cross-correlation between two boundary networks b and b'. However cross-correlation itself is not well-suited for dealing with the non-negativity of the edge weightings, so we calculate a non-centered version of it. The *absolute cross-correlation* of the two boundary networks b and b' is then given by the normalized scalar product of their edge weightings, i.e. by

$$a(b, b') := \frac{(1/|E|) \sum_{e \in E} b(e) b'(e)}{\sqrt{(1/|E|) \sum_{e \in E} b(e)^2} \sqrt{(1/|E|) \sum_{e \in E} b'(e)^2}}$$

where E denotes the set of edges connecting adjacent counties. This quantity lies between 0 and 1 and equals 1 if and only if the two boundaries are identical up to scaling.

Apart from the upper bound, this quantity however is difficult to judge. In particular, we cannot compare right away two cross-correlations between different networks since $a(\cdot, \cdot)$ might depend on the number of clusters and inhomogeneity of weights etc. We avoid finding a direct interpretation of the absolute cross-correlation by instead considering deviation of observed values against cross-correlations with a null model.

Such null models are used to tell random occurrences of structures from true information. One typically wants to keep some statistics of the network fixed while at the same time randomly sampling from its representational class. This results in the notion of random graphs with certain additional properties such as Erdös-Rényi [7] or Barabási-Albert [2]. The key idea is to generate a random network preserving planarity and possible additional information by using the original structure and iteratively changing it by a random local modification. For instance for unweighted networks, a random graph can be generated by 'rewiring': two distinct edges and two different vertices contained in either of the two are randomly selected and then swapped. Clearly this operation keeps both degree distributions fixed. After a certain number of iterations, the thus generated Markov chain produces independent samples of the underlying random graph with given degree distributions [13]. This concept has been generalized to weighted graphs [16]; in this case it is debatable whether to swap the whole weighted edge or to split up the weight.

In our case we search for a randomization of a boundary network i.e. of a planar, weighted graph. Rewiring as above is not possible since it would destroy planarity. Instead we propose to locally modify the graph at a random county: select a subpath of its boundary and flip it to its complement. In the case of non trivial weights, we reassign a random number between 0 and the minimal edge weight on the subpath. We have illustrated this procedure on an example in Fig. S20.

This procedure is now repeated multiple times until sufficiently decorrelated samples from the original network are produced. In practice, it is common to choose iterations in the range of the number of edges in the network or more.

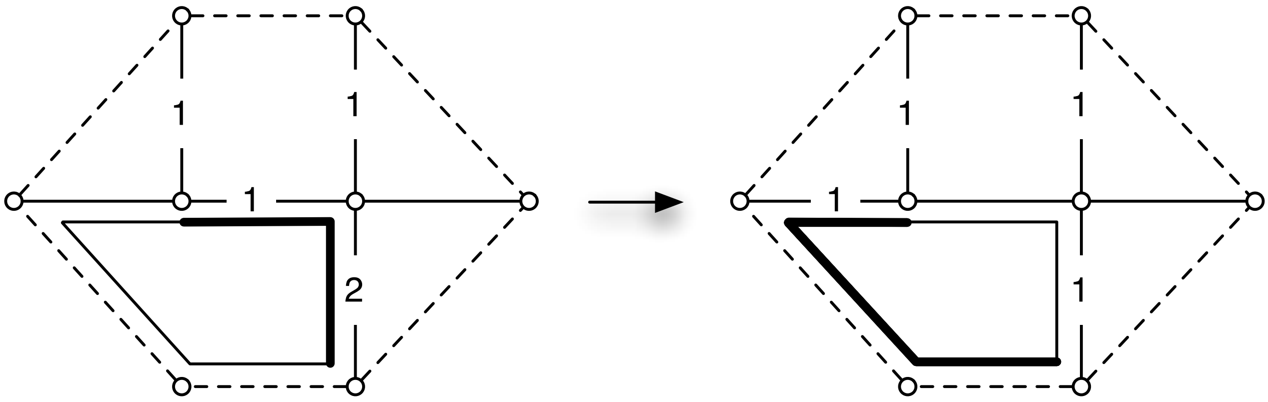

Figure S20: Local modification of a planar graph. We select the bottom left county to modify. The selected path to modify is shown in bold in the left figure. Its minimal weight is 1. This is subtracted in the right hand figure, where the complementary path is shown.

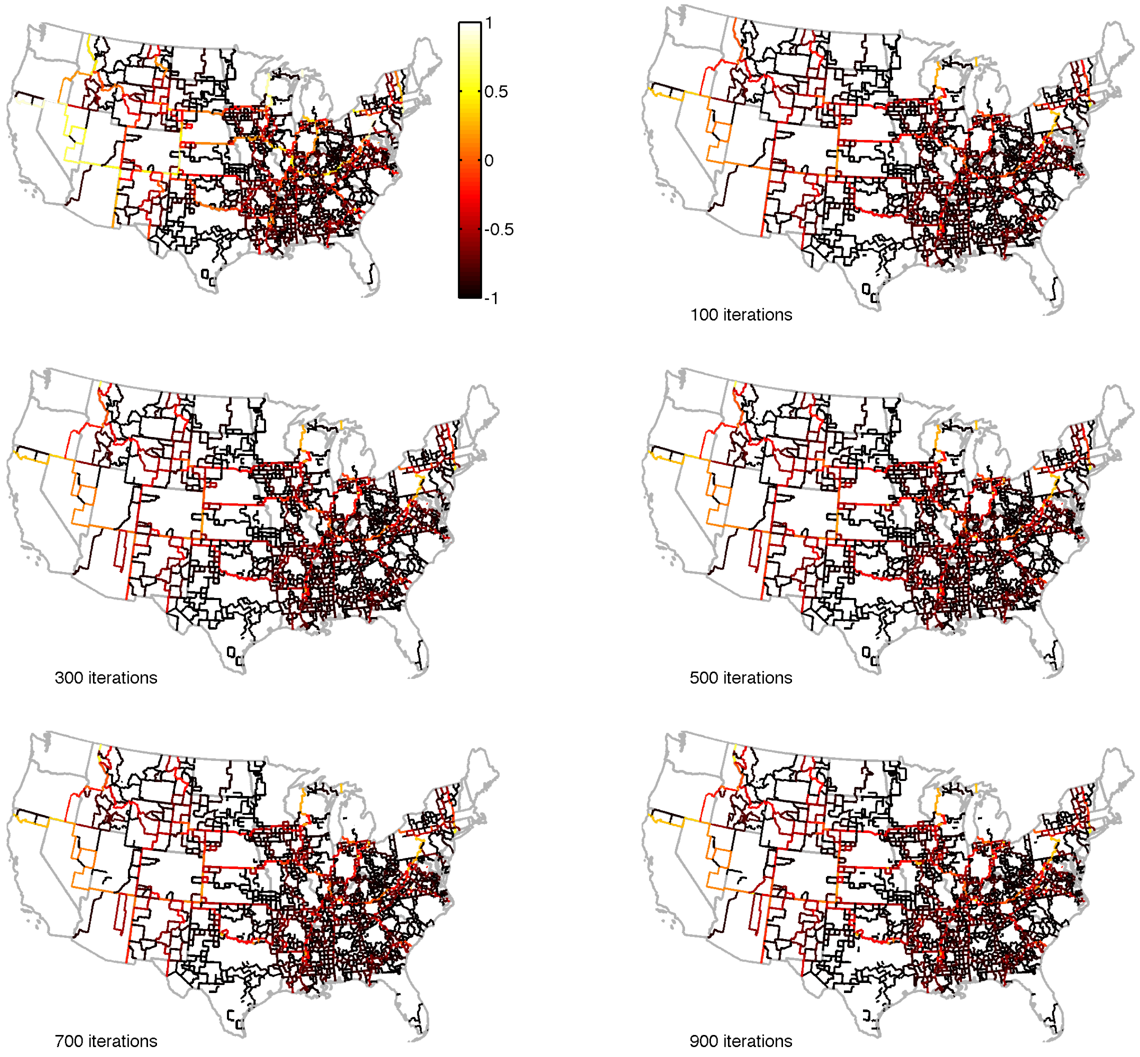

Figure S21: Randomization of the modularity boundary network b_M. The original network and the first 900 iterations are shown.

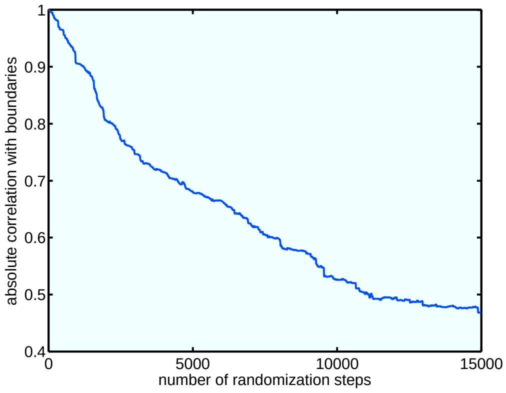

Figure S22: Absolute cross-correlation of the randomized network after the given number of iterations with the modularity boundary network b_M.

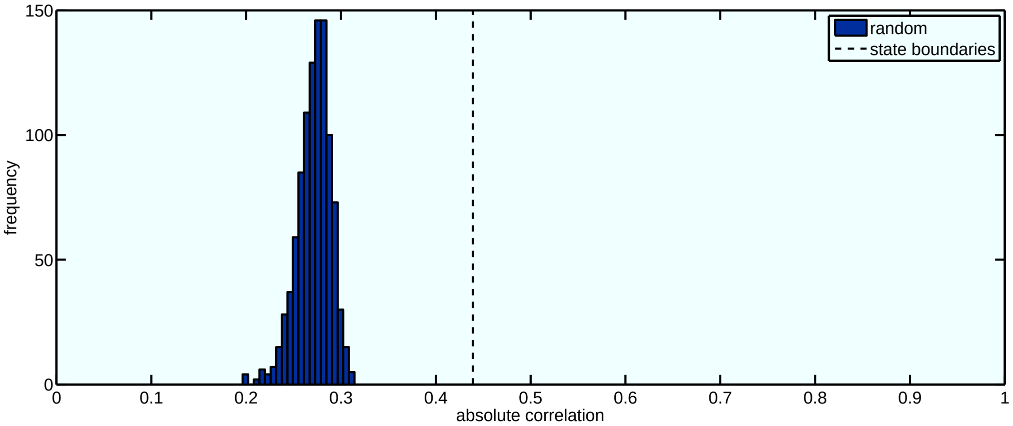

(a) absolute correlations of the null model and b_M with the state boundaries

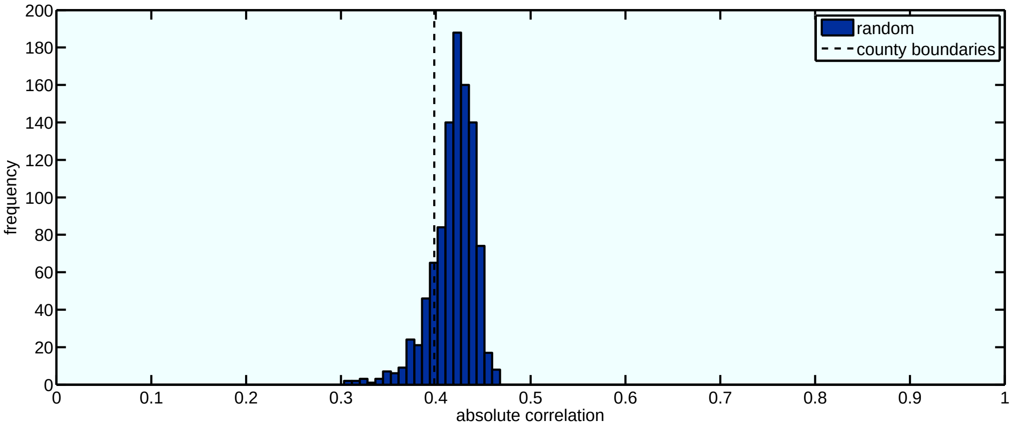

(b) absolute correlations of the null model and b_M with the county boundaries

Figure S23: Absolute cross-correlation of state and county boundaries when compared with a null model based on the modularity boundary network b_M.

6.2. Randomization of the mean partition boundary of the Where's George network

In order to test for significances of calculated similarities, we build a random model of the mean partition boundary by generating 1000 random networks using the above algorithm with > 15000 successful iterations for each random network. The corresponding maps for the first 1000 iterations are shown in Fig. S21. Clearly the original structure in the boundary network is increasingly diluted, and after > 10000 iterations becomes stably random.

This can be seen by calculating the absolute cross-correlation $a(b, b_R)$ of the modularity boundary network b_M with the random networks b_R, when increasing the number of iterations, see Fig. S22. We observe convergence to roughly 0.5 after about 10000 steps. This lies well in the range of random correlation with a mean of 0.49 and a standard deviation of 0.028, see histogram in Fig. S23(a). This implies that the randomization procedure converges to a set of random boundary networks, which can now be used to put calculated autocorrelations into perspective against this null model.

6.3. Significances when comparing boundary networks with the null model

We describe and quantify overlap of the estimated modularity boundaries b_M with other political or social boundaries. As described before, we can quantify overlap by determining the absolute cross-correlation $a(b, b_M)$. In order to determine interpretable numbers, we compare this value to correlations with random boundaries b_R from a null model.

We now determine significance of coincidence of the modularity boundary network b_M and the SPT boundary network b_S with

- modularity boundaries b_M,
- state boundaries,
- county boundaries (to test for sensitivity of the method against number of communities),
- boundaries resulting from the SPT algorithm b_S,
- boundaries determined on the gravity model,
- boundaries determined on long-range distances only,
- federal reserve district boundaries (FRB), and
- economic area boundaries (`http://www.bea.gov`).

The significance is calculated by replacing b_M and b_S, respectively, by elements from the corresponding null model.

For illustration we show two histograms and actual values for state and county boundaries in Fig. S23. Clearly the random cross-correlations are quite different, which means that we have to interpret the actual values of 0.439 and 0.398 differently as well. Indeed it turns out that the state value is far from the mean random cross-correlation 0.272 ± 0.018, whereas the county one is not (0.419 ± 0.023). Indeed, the empirical p-values, determined as the fraction of random correlations above the observed true one, is 0 in the former and 0.84 in the latter case.

In order to compare cases with large deviation from the distribution, we determine the z-score i.e. the distance of the absolute cross-correlation from the mean of the null model normalized by the standard deviation:

$$z(b) := \frac{a(b, b_M) - E(a(b, b_R))}{\mathrm{std}(a(b, b_R))},$$

where E denotes mean and std standard deviation. In the state case, this z-score is very high, 9.46, which means that the observed correlation is more than 9 standard deviations away from the random

mean. In contrast the county z-score is 0.90, which means that the observation is within one standard deviation and hence not significant.

We summarize the calculated cross-correlations in Tables S6 and S7 for b_M and b_S.

Table S6: Comparing boundary overlaps for various boundary networks with the modularity boundaries b_M and the corresponding null model b_R using absolute cross-correlation a.

boundary network	$a(\cdot, b_M)$	$a(\cdot, b_R)$	p-value	z-score
modularity boundaries	1.000	0.495 ± 0.028	$< 10^{-3}$	18.15
SPT communities	0.552	0.385 ± 0.024	$< 10^{-3}$	7.03
state boundaries	0.439	0.272 ± 0.018	$< 10^{-3}$	9.46
county boundaries	0.398	0.419 ± 0.023	0.84	0.90
gravity boundaries	0.260	0.253 ± 0.019	0.35	0.40
large-range network boundaries	0.198	0.181 ± 0.017	0.14	1.02
federal reserve district boundaries	0.377	0.227 ± 0.019	$< 10^{-3}$	7.91
economic area boundaries	0.452	0.307 ± 0.018	$< 10^{-3}$	8.024

Table S7: Comparing boundary overlaps for various boundary networks with the SPT-based boundary b_S and the corresponding null model b_R using absolute cross-correlation a.

boundary network	$a(\cdot, b_S)$	$a(\cdot, b_R)$	p-value	z-score
modularity boundaries	0.552	0.251 ± 0.013	$< 10^{-3}$	22.55
SPT communities	1.000	0.367 ± 0.0164	$< 10^{-3}$	40.63
state boundaries	0.358	0.220 ± 0.0138	$< 10^{-3}$	10.99
county boundaries	0.569	0.562 ± 0.016	0.36	0.44
gravity boundaries	0.305	0.260 ± 0.016	0.002	2.73
large-range network boundaries	0.257	0.199 ± 0.015	$< 10^{-3}$	3.94
federal reserve district boundaries	0.307	0.159 ± 0.013	$< 10^{-3}$	11.79
economic area boundaries	0.492	0.318 ± 0.013	$< 10^{-3}$	13.29

6.4. Discussion

For the state and the SPT boundaries we observe a strong deviation from the null model when comparing against the modularity boundaries. So we can conclude that both state boundaries and SPT boundaries are more similar to b_M than expected by chance with a p-value $< 10^{-3}$.

This is not the case for the gravity model, the county boundaries and the long-range model. In these cases, the cross-correlation with b_M is not larger than with a random model (p-value ≈ 0.44, ≈ 0.84 and ≈ 0.14). This means that they do not significantly coincide with b_M.

The absolute cross-correlation of the FRB boundaries with b_M is $a(b_F, b_M) = 0.38$, which is significantly high when compared with the null model, which exhibits cross-correlations of only $a(b_F, b_R) = 0.23 \pm 0.019$. We observe a strong deviation from the null model and can therefore conclude that the FRB boundaries are more similar to b_M than expected by chance with a p-value $< 10^{-3}$.

The corresponding z-score equals 7.91, which is lower than the one for states (9.46). This implies that the modularity boundaries' overlap with the states is larger than the one with the FRB boundaries.

We interpret the results on the FRB boundaries when compared with b_M as follows:

- The structure of b_M may be (partially) due to political structure i.e. result from b_S or due to additional money transport within FRB districts i.e. correlate with b_F. Since both b_S and b_F share strong similarties, in each of the two situations, we would see overlap with both boundaries, so we can only judge strength of overlap with respect to the other boundary.

- We quantified strength of overlap by deviation from the null model, and the corresponding z-score was more than 1.5 standard deviations higher for the state model. This stronger overlap of states with b_F therefore favors the first hypothesis i.e. the situation that political boundaries are a stronger factor for the pattern observed in b_M. In the case of dominance of the second hypothesis, we would instead expect to still see overlap with state boundaries, but less overlap than with the FRB ones.

A. Global network properties

The following table lists the connectivity (ratio of existing links and possible links in the network), and number of links L and total flux F relative to the respective quantities in the full mobility network (L_0 and F_0), the mean outreach $\bar{d} = \langle d_i \rangle$ of all nodes (with outreach being defined as the weighted average distance of all links of a node, $d_i = \sum_j w_{ij}\, d_{ij} \,/\, \sum_j w_{ij}$), the mean maximum link distance $\bar{d}^m = \langle d_i^m \rangle$ (with $d_i^m = \max_j\{d_{ij}\}$), as well as the mean $\bar{Q}$ and standard deviation σ_Q of the modularity values, and the minimum ($k_{\min}$), median ($\bar{k}$), and maximum ($k_{\max}$) number of modules found in the ensemble of N_P partitions:

	Mobility network	Long-range subnetwork	Gravity model network
Connectivity	0.0765	0.0511	0.0762
L/L_0	1	0.668	0.996
F/F_0	1	0.219	1.159
$\bar{d}$ (in km)	451	1,135	808
$\bar{d}^m$ (in km)	3,079	3,079	3,099
N_P	1000	329	500
$\bar{Q}$	0.6744	0.2180	0.4791
σ_Q	0.0026	$9.01 \cdot 10^{-6}$	0.0044
$k_{\min}$	11	3	5
$\bar{k}$	13	3	6
$k_{\max}$	16	3	7

References

[1] E. G. Altmann, J. B. Pierrehumbert, and A. E. Motter. Beyond word frequency: Bursts, lulls, and scaling in the temporal distributions of words. *PLoS ONE*, 4(11):e7678, 11 2009.

[2] A. Barabási and R. Albert. Emergence of scaling in random networks. *Science*, 286:509–512, 1999.

[3] A.-L. Barabási. The origin of bursts and heavy tails in human dynamics. *Nature*, 435:207, 2005.

[4] D. Brockmann, L. Hufnagel, and T. Geisel. The scaling laws of human travel. *Nature*, 439(7075):462–465, 2006.

[5] L. Danon, A. Díaz-Guilera, J. Duch, and A. Arenas. Comparing community structure identification. *Journal of Statistical Mechanics*, 9:P09008, 2005.

[6] E. Dijkstra. A note on two problems in connexion with graphs. *Numerische Mathematik*, 1(1):269–271, Jan 1959.

[7] P. Erdos and A. Rényi. On random graphs. i. *Publicationes Mathematicae*, 6:290–297, 1959.

[8] B. Everitt, S. Landau, and M. Leese. *Cluster analysis.* Jan 2001.

[9] S. Fortunato and M. Barthélemy. Resolution limit in community detection. *Proceedings of the National Academy of Sciences*, 104:36–41, jan 2007.

[10] M. C. González, C. A. Hidalgo, and A.-L. Barabási. Understanding individual human mobility patterns. *Nature*, 453(7196):779–782, 2008.

[11] B. H. Good, Y.-A. de Montjoye, and A. Clauset. The performance of modularity maximization in practical contexts. *arXiv Preprint Server*, page 0910.0165v1, 2009.

[12] R. Guimerà and L. A. N. Amaral. Cartography of complex networks: modules and universal roles. *Journal of Statistical Mechanics*, 2:P02001, 2005.

[13] S. Maslov and K. Sneppen. Specificity and stability in topology of protein networks. *Science*, 296(5569):910–3, May 2002.

[14] M. E. J. Newman and M. Girvan. Finding and evaluating community structure in networks. *Physical Review E*, 69(2):026113, Feb. 2004.

[15] R. Sokal and F. Rohlf. The comparison of dendrograms by objective methods. *Taxon*, 11(2):33–40, Jan 1962.

[16] V. Zlatic, G. Bianconi, A. Díaz-Guilera, D. Garlaschelli, F. Rao, and G. Caldarelli. On the rich-club effect in dense and weighted networks. *European Physical Journal B*, 67:271–275, 2009.
